# Blood-Based Biomarkers in Alzheimer’s Disease: Advancing Non-Invasive Diagnostics and Prognostics

**DOI:** 10.3390/ijms252010911

**Published:** 2024-10-10

**Authors:** Mrinmay Dhauria, Ritwick Mondal, Shramana Deb, Gourav Shome, Dipanjan Chowdhury, Shramana Sarkar, Julián Benito-León

**Affiliations:** 1 Regional Centre for Biotechnology, Faridabad 121001, India; mrinmay.dhauria@gmail.com; 2 Department of Clinical Pharmacology and Therapeutic Medicine, IPGMER and SSKM Hospital, Kolkata 700020, India; ritwickraw@gmail.com; 3 Department of Stroke Medicine, Institute of Neuroscience, Kolkata 700017, India; shramanadeb1995@gmail.com; 4 Department of Biological Sciences, Bose Institute, Kolkata 700054, India; gshome007@gmail.com; 5 Department of Internal Medicine, IPGMER and SSKM Hospital, Kolkata 700020, India; dipanjan.1729@gmail.com (D.C.); shramana99.sarkar@gmail.com (S.S.); 6 Department of Neurology, University Hospital “12 de Octubre”, ES-28041 Madrid, Spain; 7 Instituto de Investigación Sanitaria Hospital 12 de Octubre (imas12), ES-28041 Madrid, Spain; 8 Centro de Investigación Biomédica en Red Sobre Enfermedades Neurodegenerativas (CIBERNED), ES-28029 Madrid, Spain; 9 Department of Medicine, Complutense University, ES-28040 Madrid, Spain

**Keywords:** Alzheimer’s disease (AD), blood-based biomarkers, amyloid-beta (Aβ), phosphorylated tau (p-tau), neurofilament light chain (NfL), non-invasive diagnostics, neurodegeneration, abnormal protein accumulation, neuroinflammation, vascular pathology, early detection, prognostics, advanced neuroimaging, dementia

## Abstract

Alzheimer’s disease (AD), the most prevalent form of dementia, is expected to rise dramatically in incidence due to the global population aging. Traditional diagnostic approaches, such as cerebrospinal fluid analysis and positron emission tomography, are expensive and invasive, limiting their routine clinical use. Recent advances in blood-based biomarkers, including amyloid-beta, phosphorylated tau, and neurofilament light, offer promising non-invasive alternatives for early AD detection and disease monitoring. This review synthesizes current research on these blood-based biomarkers, highlighting their potential to track AD pathology and enhance diagnostic accuracy. Furthermore, this review uniquely integrates recent findings on protein-protein interaction networks and microRNA pathways, exploring novel combinations of proteomic, genomic, and epigenomic biomarkers that provide new insights into AD’s molecular mechanisms. Additionally, we discuss the integration of these biomarkers with advanced neuroimaging techniques, emphasizing their potential to revolutionize AD diagnostics. Although large-scale validation is still needed, these biomarkers represent a critical advancement toward more accessible, cost-effective, and early diagnostic tools for AD.

## 1. Background

Neurodegeneration in Alzheimer’s disease (AD) is associated with toxic amyloid-beta (Aβ) oligomers, protein aggregates, intra-neuronal neurofibrillary tangles consisting of hyperphosphorylated microtubule-associated tau protein, synaptic dysfunction, reduced cerebral glucose metabolism, and mitochondrial dysfunction [1]. AD accounts for more than 50–70% of cases among all neurodegenerative dementias, and it is estimated that approximately 44 million people worldwide are living with AD dementia, a number that could triple by 2050 [2]. Only a tiny percentage (1%) of AD is inherited, known as early-onset AD (EOAD). Most cases are sporadic and generally appear after age 65, also known as late-onset AD [3]. The age of onset for late-onset AD can vary between countries, typically occurring at 65 in the USA, whereas in India, it generally develops after 60 years of age [3]. In addition to genetic factors, other contributors such as reduced physical activity, poor diet, diabetes, cerebrovascular disease, and stress are major risk factors in disease progression.

According to the 2018 National Institute on Aging–Alzheimer’s Association (NIA-AA) framework, AD should be considered in a biological context rather than a syndromic one, using an A/T/N classification system. In this system, “A” represents the concentration of Aβ biomarkers, “T” indicates the level of tau, and “N” reflects biomarkers of neurodegeneration [4]. This categorization prioritizes the classification of AD biomarkers according to pathological mechanisms. However, this framework assumes equivalence between cerebrospinal fluid (CSF) and imaging biomarkers within each AT(N) category [5], an assumption that is not always supported by evidence. The AD diagnosis can be strengthened by including additional biomarkers that reflect changes in brain vascularity, Lewy body pathology, and neuroinflammation [5].

In recent years, the understanding of AD has shifted from diagnosing and characterizing the disease based on clinical presentation alone to a biologically driven approach [6,7]. This transition emphasizes the importance of disease staging through research and clinical care. However, the high cost of positron emission tomography (PET) and the invasiveness of CSF sampling are significant obstacles to population-wide screening for early, potentially manageable AD [8]. Minimally invasive approaches, such as blood-based and fluid biomarkers, show promise in revolutionizing the diagnostic and prognostic workflow in clinical settings, particularly with the recent introduction of anti- Aβ immunotherapies. Standardizing plasma-based assays and integrating non-invasive neuroimaging techniques could reduce reliance on invasive procedures, such as CSF sampling or PET scans, making AD diagnostics more accessible and cost-effective [9].

This review contributes to the field by uniquely integrating recent advancements in blood-based biomarker (BBBM) research, particularly in the context of protein-protein interaction (PPI) networks and microRNA pathways. Unlike previous reviews, this work explores novel combinations of proteomic, genomic, and epigenomic biomarkers to uncover new insights into AD’s underlying molecular mechanisms. Additionally, this review highlights the clinical applicability of these biomarkers, combining their diagnostic accuracy with advanced neuroimaging techniques. By focusing on minimally invasive, scalable diagnostic tools, this review offers a comprehensive framework that has the potential to enhance early detection, staging, and treatment strategies for AD, especially in resource-limited settings.

## 2. Methodology

We conducted a comprehensive search of PubMed, Embase, and Google Scholar using the following search terms: “Alzheimer’s disease”, “Alzheimer’s disease and related dementia”, “plasma”, “blood”, “serum”, and “biomarkers.” The search was limited to articles published in English in the last ten years. Our primary focus was on studies evaluating BBBMs for AD diagnosis, with a specific emphasis on plasma biomarkers.

To ensure completeness, we also reviewed reference lists of the selected articles to identify any relevant studies that may have been missed in the initial search. The inclusion criteria for this review emphasized studies that directly examined BBBMs linked to AD, their diagnostic accuracy, and their correlation with established CSF and neuroimaging biomarkers.

We enhanced our analysis by employing advanced bioinformatics tools to explore PPI and microRNA network analyses. We utilized the Search Tool for the Retrieval of Interacting Genes/Proteins (STRING) database version 12.0 (https://string-db.org/ (accessed on 25 August 2024) to generate PPI networks. Query proteins were uploaded using their gene symbols obtained from the National Center for Biotechnology Information (NCBI). The resulting PPI network illustrated both functional and physical interactions among the query proteins. Network edges were supported by multiple lines of evidence, including text mining, co-expression data, curated databases, gene neighborhoods, gene fusions, co-occurrence, and protein homology. To ensure robustness, we maintained a high confidence interaction score threshold (≥0.700).

Additionally, we used the miRNeT 2.0 database (https://www.mirnet.ca/ (accessed on 28 August 2024) to analyze the interaction network of predicted AD-related microRNA biomarkers with validated target genes. This network was visualized to prioritize key microRNA biomarkers and their roles in gene regulation within both peripheral blood and brain tissues. For clustering, we set parameters such as organism (Homo sapiens), ID type (miRBase ID), tissue types (peripheral blood and brain), and target database (miRTarBase v9.0). Network topology features such as degree and betweenness centrality were used to analyze microRNA-microRNA interactions further.

## 3. Results/Discussion

### 3.1. Current Insights into Different Biomarker Categorizations

The development of in vivo biomarkers has shifted the diagnosis of AD from the late or advanced dementia stages of the disease to earlier stages. It has introduced the potential for pre-symptomatic diagnosis. Categorization of biomarkers involves grouping them into categories that reflect shared pathways of abnormal protein accumulation or underlying disease processes. According to recent recommendations by the Alzheimer’s Association workgroup, AD biomarkers can be broadly categorized into the following [7]:

1. Core biomarkers of AD neuropathological changes;
2. Non-specific biomarkers that are important in AD pathogenesis but are also involved in other brain diseases;
3. Biomarkers of common non-AD pathologies.

This broad categorization and sub-categorization are based on specific pathways of abnormal protein accumulation or pathogenic sequences. Importantly, imaging biomarkers reveal cumulative effects and provide topographic data aligned with established neuropathological constructs. In contrast, blood or fluid-based biomarkers generally reflect the dynamic balance between the production and clearance of specific analytes at a given time point.

**Figure 1:**
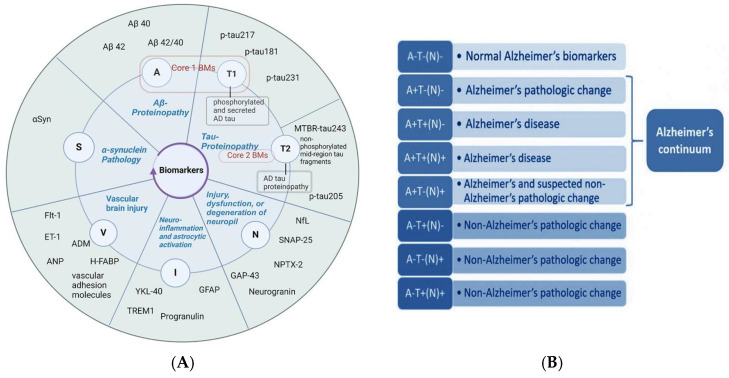
(**A**): From ATN to AT1T2NIVS biomarker categorization of fluid analytes: The proposed new criteria by the NIA-AA 2024 working group emphasize ‘A’ and ‘T’ as the core biomarkers for the diagnosis and staging of AD. In addition, the revised scheme recognizes an expanded set of additional markers that detect non-specific biomarkers involved in AD pathophysiology (categorized under ‘N’ and ‘I’) and non-AD co-pathological biomarkers (categorized under ‘V’ and ‘S’). The core biomarkers are further divided into Core 1 and Core 2 biomarkers to reflect different stages of AD-related changes. (**B**): This figure depicts the biomarker profile and corresponding categorization based on the “A”, “T”, and “N” systems. By binarizing the three AT(N) biomarker types, eight distinct biomarker profiles are generated. Based on these profiles, individuals can be placed into one of three general categories: standard AD biomarkers, the Alzheimer’s continuum, or non-AD pathological changes.

The recent updates, as per the international working group, incorporate recently developed BBBMs of “A”, “T”, and (N) [6,7]. Core AD biomarkers, according to recent updates, fall within the “A” (Aβ) and “T” (tau) categories. The “A” category represents biomarkers associated with the abnormal accumulation of Aβ; it is well known that soluble aggregation-prone Aβ peptides are the essential building blocks of insoluble Aβ fibrillary aggregates in plaques. These peptides reflect the temporal dynamics of different biochemical pools in the abnormal protein accumulation pathway, as detected through fluid-based assays and imaging techniques [7] [Figure 1A].

Accordingly, “T” biomarkers also exhibit varying temporal relationships across the spectrum. These differences can be categorized into subcategories: T1 and T2. T1 represents tau PET imaging or biofluid analytes of soluble tau fragments that react to amyloid plaques or soluble Aβ species in the plaque penumbra. T2 refers to tau PET imaging or biofluid analytes that indicate the presence of AD tau aggregates. Consequently, Core 1 biomarkers (A and T1) define the initial stage of AD as detectable in vivo and capable of identifying AD in symptomatic and asymptomatic individuals.

In contrast, Core 2 biomarkers (T2 category) include tau-PET and specific soluble tau fragments related to tau accumulation, which generally reflect more advanced stages of AD pathogenesis. Hence, Core 2 biomarkers do not detect the initial presence of the disease and are highly associated with Aβ pathology. Therefore, Core 2 biomarkers, combined with Core 1, may be used to stage the biological disease severity [7].

In general, AD biomarkers can be classified into four major categories:

[A] Diagnostic markers: PET imaging and CSF analysis for Aβ and tau proteins are well-established diagnostic tools. PET imaging visualizes the accumulation of Aβ plaques and tau tangles in the brain, while CSF analysis measures concentrations of Aβ42, total tau (t-tau), and phosphorylated tau (p-tau), providing direct biochemical evidence of AD pathology.

[B] BBBMs: Non-invasive BBBMs, such as phosphorylated tau (pTau), Aβ42/40 ratio, and neurofilament light chain (Nfl), are gaining importance as they correlate well with CSF and PET findings. Additionally, microRNA signatures related to neurodegeneration are being researched for their potential diagnostic utility. Although still in development, these markers offer a promising and accessible alternative to invasive tests.

[C] Fluid-based biomarkers: Saliva and urine are being investigated for their potential to serve as AD biomarkers, but their diagnostic reliability is not yet established. While they offer non-invasive collection methods, their use remains mainly experimental, and further validation is needed before they can be considered clinically reliable.

[D] Non-invasive neuroimaging techniques: This category includes both structural and functional neuroimaging tools that provide critical insights into AD pathology. Fundamental techniques include magnetic resonance imaging (MRI) (both structural and functional), diffusion tensor imaging, functional near-infrared spectroscopy, magnetoencephalography, single-photon emission computed tomography, and magnetic resonance spectroscopy.

These imaging methods offer complementary structural, functional, and neurochemical information. For example, MRI and PET imaging provide detailed topographical data on Aβ plaque and tau tangle distribution, while techniques like functional MRI and magnetoencephalography assess functional connectivity and brain activity.

Non-invasive neuroimaging approaches, such as functional MRI (fMRI) or PET imaging, offer valuable structural and functional insights into AD progression. While neuroimaging alone cannot provide a definitive AD diagnosis, combining it with BBBMs (e.g., p-tau, Nfl) significantly enhances diagnostic accuracy. Neuroimaging visualizes the distribution of pathological changes, while blood biomarkers provide molecular-level insights into AD pathophysiology. Together, these methods form a robust framework for early detection and continuous monitoring of disease progression, particularly in resource-limited settings where invasive tests like CSF analysis or PET scans may not be feasible.

### 3.2. The Context for Developing BBBMs in AD

The development of BBBM for AD has become essential in both research and clinical settings. These biomarkers play a crucial role in understanding the disease’s etiopathogenesis, as well as in monitoring disease progression and assessing therapeutic efficacy. BBBMs facilitate faster clinical decision-making, optimize healthcare resources, and enhance overall efficiency. Additionally, they are critical for population screening to detect AD early and to identify participants eligible for clinical trials [10].

Adopting BBBMs has significant public health benefits, particularly in middle- and low-income countries, where access to invasive diagnostics (like PET scans or CSF analysis) may be limited. Their versatility allows ongoing research and innovation to be effectively utilized, ultimately improving outcomes for individuals diagnosed with AD.

Furthermore, BBBM tests, which provide a non-invasive alternative to CSF analysis or PET scans, are valuable for diagnosing AD in symptomatic patients with cognitive impairment. These biomarkers can also guide therapeutic decisions, facilitating personalized treatment and disease management. In resource-limited clinical settings, where CSF analysis or PET scans may not be feasible, blood-based screening offers a practical solution for evaluating patients with dementia [11].

**Figure 2:**
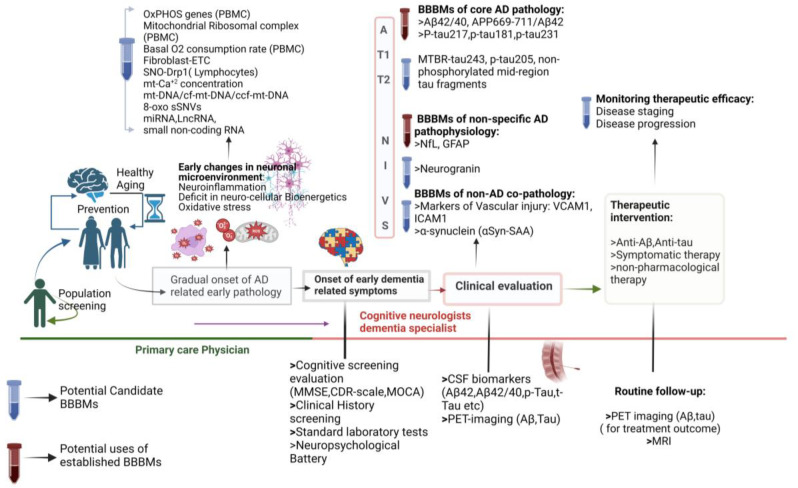
Timeline of patients’ journey integrated with blood-based biomarkers (BBBM). The figure illustrates the patient journey, starting with population screening and preventive strategies aimed at healthy aging. As AD-related pathology develops, BBBMs such as Aβ42/40, p-tau, and NfL are introduced during visits to a primary care physician for cognitive screening and early detection. Patients are then referred to specialists (neurologists or dementia experts) for clinical evaluation, which integrates BBBMs, CSF biomarkers, and PET imaging to confirm diagnosis. Biomarkers are also used for therapeutic intervention and monitoring disease progression, supporting treatment strategies like anti-Aβ or anti-tau therapies.

Looking ahead, blood-based screening holds great promise for identifying AD in asymptomatic individuals, which could enable early interventions to slow the onset and progression of dementia potentially. This would reduce the overall disease burden and improve long-term outcomes. Such an approach promotes early detection, accurate diagnosis, and personalized therapeutic interventions, elevating AD management to a state-of-the-art level [Figure 2]. Ultimately, large-scale validation studies are necessary to establish the reliability, sensitivity, and specificity of BBBM across diverse populations, improving AD diagnosis and patient stratification based on disease severity and progression in clinical settings.

### 3.3. BBBMs Related to Abnormal Protein Accumulation for the Early Detection of AD

**Figure 3:**
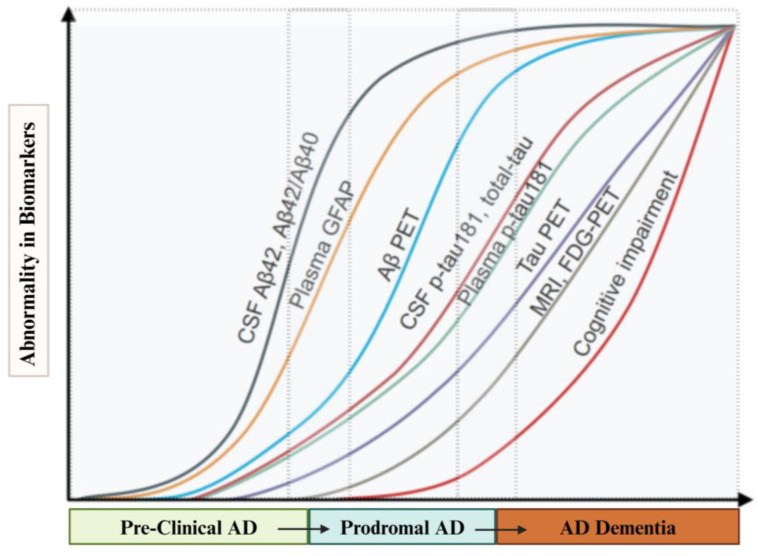
An approximate relationship between biomarker abnormality and the Alzheimer’s disease (AD) continuum (pre-clinical, prodromal, and AD dementia). The figure demonstrates the progressive increase in biomarker abnormalities across the AD continuum. In the pre-clinical phase, changes in CSF Aβ42, Aβ42/Aβ40 ratio, and plasma GFAP occur early. As the disease progresses to the prodromal phase, abnormalities in Aβ PET, CSF p-tau181, total-tau, and plasma p-tau181 become evident. In the later stages, including AD dementia, abnormalities in tau PET, MRI/FDG-PET, and cognitive impairment markers become pronounced. The figure illustrates the evolving nature of these biomarkers in the context of AD progression.

Research has demonstrated that proteins expressed in brain tissues can be detected in peripheral circulation. These proteins, associated with abnormal protein accumulation, are becoming critical tools for identifying AD-related pathology. Several studies have established connections between plasma biomarkers related to abnormal protein accumulation and corresponding PET imaging, CSF biomarkers, and cognitive staging [12] [Figure 3], emphasizing the increasing importance of BBBM in AD diagnostics and research.

#### 3.3.1. Plasma Biomarkers Related to Abnormal Protein Accumulation as Core Indicators of AD

Core AD-related biomarkers in plasma are categorized into A (Aβ) and T (tau). The A category includes biomarkers associated with Aβ accumulation, reflecting different stages of Aβ aggregate formation during the disease process. The T category encompasses biomarkers that indicate the timing and progression of various modified forms of tau proteins, which are essential for tracking disease advancement.

##### Aβ and Its Variations in Plasma

Identifying and validating accurate and reliable BBBM for Aβ accumulation has been highly challenging. Plasma or serum Aβ42 levels are 10–100 times lower than their CSF counterpart, and Aβ structural epitopes can be masked due to their binding affinity with plasma proteins [13]. Additionally, the variable peripheral sources of Aβ pose challenges in obtaining reliable and consistent measurements of Aβ in peripheral circulation across different laboratories and study cohorts when using conventional enzyme-linked immunoassay (ELISA) [14]. However, recent advances in peripheral Aβ measurement, including cutting-edge techniques such as immunomagnetic reduction, single-molecule array (SIMOA), immunoprecipitation, and liquid chromatography-mass spectrometry, have significantly improved the accuracy and standardization of peripheral Aβ levels in AD across various laboratories [15,16,17]. In a head-to-head study involving ten different assays, the liquid chromatography-mass spectrometry method demonstrated the best diagnostic performance among all tested assays [17].

Notably, the advancement of SIMOA has enabled the measurement of Aβ with high precision. It has demonstrated the ability to accurately measure plasma Aβ40/Aβ42 levels, effectively predicting amyloid-positive PET scans in both cognitively normal and impaired individuals. [13]. However, the study by Blennow and Zetterberg et al. [18] revealed that AD patients with pathological CSF signatures showed significant differences in plasma Aβ42 from control, indicating a limited potential of plasma Aβ for distinguishing pre-clinical AD with CSF pathologies.

Interestingly, a study by Guo et al. [19] demonstrated different dynamic trends throughout the AD continuum. The plasma Aβ42/40 levels were significantly reduced in the cognitively unimpaired A+T+ group compared to the cognitively unimpaired A-T- group. Similar trends were observed between the mild cognitive impairment (MCI) or AD dementia group and the cognitively unimpaired A-T- group. Additionally, the AD dementia group showed reduced plasma Aβ42/40 levels relative to the cognitively unimpaired A+T- and MCI+ groups. However, individual plasma levels of Aβ42 and Aβ40 remained unchanged, except for increased Aβ40 levels in the AD dementia group compared to the cognitively unimpaired A-T- group [19].

Plasma composite biomarkers, including normalized scores for amyloid precursor protein (APP)669–711/Aβ1–42 and Aβ1–40/Aβ1–42, have shown a strong correlation with CSF levels, achieving 80.4% accuracy in patients with AD. This association performs comparably to CSF Aβ42 in determining brain Aβ burden. Using immunoprecipitation mass spectrometry, Nakamura et al. [20] demonstrated that plasma Aβ predicted brain Aβ burden more accurately than classification using Aβ PET. Additionally, Schindler et al. [21] found that the plasma Aβ42/40 ratio, combined with age and Apolipoprotein E status, achieved high diagnostic accuracy for brain amyloidosis using a liquid chromatography-mass spectrometry technique. In 2017, Ovod et al. [22] showed that the plasma Aβ42/40 ratios, measured by liquid chromatography-mass spectrometry, had an area under the receiver operating characteristic curve (AUC) of 0.88, in differentiating amyloid positivity in Aβ-PET or CSF. The relationship between Aβ biomarker abnormalities and AD progression is illustrated in Figure 3.

However, there are significant challenges to the widespread use of plasma Aβ as a surrogate measure of brain amyloid pathology. Notably, the differences in plasma Aβ levels between Aβ-PET (+) and Aβ-PET (−) groups are only around 10–15%, compared to 40–60% when measured in CSF [23]. Therefore, combining biomarkers often improves the overall accuracy of Aβ measurement. A better understanding of cohort differences, sample processing procedures, and the influence of other AD risk factors has enhanced the diagnostic properties of plasma Aβ assays.

For instance, using the clinically available PrecivityAD™ test, a liquid chromatography-mass spectrometry method with a plasma Aβ42/40 ratio cut-off value of 0.0975, an AUC of 0.81, and an accuracy of 75% was achieved. After adjusting for cohort differences, the AUC increased to 0.86 and the accuracy to 81%. With additional adjustments for age and Apolipoprotein E status, the AUC improved further to 0.90, with an accuracy of 86%. Notably, the diagnostic accuracy of this method was not significantly affected by potential confounding variables, such as variations in plasma sample collection across different cohorts [24]. 

##### Plasma p-Tau

In recent updates by the NIA-AA working group, various subtypes of Tau have been classified into the T1 (Core-1) and T2 (Core-2) subcategories within the core AD-related pathological markers. T1 represents early tau changes, including p-tau proteins, while T2 reflects more advanced stages of tau accumulation, which is strongly associated with disease progression in AD [7]. The fundamental concepts of Core-1 and Core-2 AD biomarkers in these recent updates are distinguished by the timing of cognitive abnormality onset. The Core-1 category represents the initial stage of AD neuropathological changes in vivo, observed in both symptomatic and asymptomatic individuals. In contrast, Core-2 biomarkers are more closely linked to Aβ pathology.

Consequently, tau accumulation serves as a more precise biomarker for cognitive decline and is strongly associated with underlying AD pathology. It also predicts the risk of future dementia in individuals with MCI [25]. However, CSF t-tau is considered a non-specific marker, as elevated levels can also be found in conditions like traumatic brain injury and acute stroke, making it more indicative of neuronal injury than AD-specific pathology.

Tau’s physiological role is to stabilize microtubules in the axons of neurons. The degeneration of neuraxial structures leads to increased Tau release from neuronal components, contributing to the disruption of normal cellular function in neurodegenerative diseases like AD [26]. Additionally, Tau undergoes truncation and subsequent phosphorylation, which leads to neurofibrillary tangle aggregation in the proximal axoplasm. Abnormal phosphorylation and truncation of the Tau protein are the primary causes of neurofibrillary tangle formation in AD and other tauopathies.

The Tau protein contains multiple phosphorylation sites. T1 represents ‘phosphorylated and secreted tau’ (pTau217, pTau181, pTau231), while T2 corresponds to ‘AD-related tau accumulation’ (microtubule-binding region tau243, pTau205, and non-phosphorylated mid-region tau fragments) [7]. Similar to plasma Aβ, a significant challenge in developing plasma-based Tau assays is the significantly lower concentration of Tau in the blood compared to CSF. The CSF Tau level is approximately 2–300 pg/mL, whereas the plasma concentration is about 100-fold lower, around 5 pg/mL.

Significant progress has been made in developing highly sensitive assays, particularly MS-based techniques, which have greatly improved the identification and quantification of plasma p-Tau. Plasma pTau181 is strongly correlated with Aβ-PET and CSF pTau181 levels, and it demonstrates high specificity in differentiating AD from other tauopathies [27,28]. Additionally, plasma pTau181 has been shown to distinguish between Aβ-PET (+) and Aβ-PET (−) individuals, as well as track disease progression to dementia and tau accumulation in brain regions associated with AD-related atrophic changes [29].

In this context, T1-related plasma-tau variations (pTau217, pTau181, pTau231) are significantly higher in AD patients compared to cognitively unimpaired individuals. Notably, a study by Guo et al. [19] demonstrated the dynamic trend of plasma pTau181 throughout the disease continuum [Figure 1B]. Plasma pTau181 levels increased in cognitively unimpaired A+T+ individuals compared to A-T- ones. A similar trend was observed in the MCI+ and AD dementia groups compared to the A-T- group. Interestingly, only pTau181 levels were higher in the cognitively unimpaired A+T+ and MCI+ groups compared to the A+T- group.

Furthermore, plasma pTau217 has shown greater diagnostic precision than pTau181 in both CSF and plasma, accurately predicting the progression from subjective cognitive decline and MCI to dementia when combined with other risk factors [30]. Ashton et al. [31], using the SIMOA-based assay, revealed that plasma pTau231, like CSF pTau217, could distinguish between patients with and without AD pathology during post-mortem assessment with an AUC of 0.99.

Additionally, a study from the BioFINDER cohort reported that plasma pTau217 could predict the progression from MCI to AD within four years, with an AUC of 0.83. The diagnostic accuracy improved further (AUC = 0.91) when plasma pTau217 was combined with the Apolipoprotein E genotype [32]. Janelidze et al. [33] also found that plasma pTau217 was significantly elevated before tau-PET became positive in cognitively unimpaired Aβ-PET+ older individuals. These findings highlight the growing potential of plasma pTau isoforms as accurate and reliable biomarkers, reflecting CSF status and predicting disease progression in AD patients. Furthermore, a review by Antonioni et al. [34] emphasized the value of blood pTau measurement for the early identification of patients within the AD continuum, noting that most studies found a correlation between CSF and blood pTau levels while also highlighting the advantages of blood pTau as a less invasive and more accessible alternative.

Interestingly, studies by González-Ortiz et al. [35] on brain-derived tau from the plasma of AD individuals have shown that it outperforms plasma t-tau. Unlike Nfl, brain-derived tau has demonstrated better specificity for AD-related neurodegeneration, indicating its potential to complete the blood AT(N) framework as a valuable biomarker for evaluating AD-dependent neurodegeneration. Notably, plasma Core 2 biomarkers, especially those in the T2 category—such as specific soluble tau fragments associated with tau accumulation and non-phosphorylated tau fragments—are still in the pre-clinical development phase [36].

Given these advancements, it is remarkable that plasma p-tau estimation can precisely diagnose AD based on clinical and pathological criteria. Additionally, it can identify individuals in the early stages of AD and monitor the pathological continuum in individuals at higher risk of cognitive decline. However, the significant variability in plasma p-tau measurement across different analytical platforms and the absence of universally accepted biomarker cut-off values limit its widespread clinical use.

**Figure 4:**
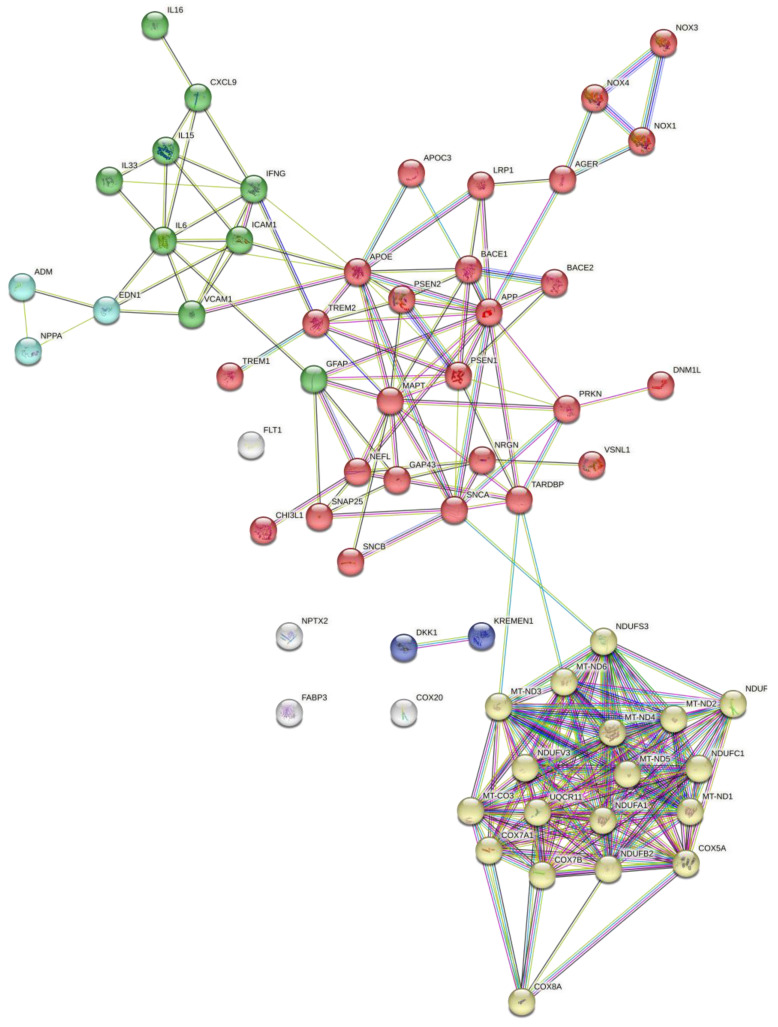
The STRING protein-protein interaction (PPI) network for AD-related blood-based biomarkers. The STRING database was queried with a subset of proteins relevant as blood biomarkers in AD, generating four distinct clusters: core AD proteins, mitochondrial OXPHOS proteins, neuroinflammatory proteins, and vascular pathology-related proteins. The PPI network highlights significant overlaps and interactions between pathways common to both blood and brain cells, reflecting the complex molecular mechanisms driving AD pathogenesis.

Table 1 summarizes various BBBM related to Aβ and p-tau accumulation. Our STRING network model highlights strong interactions between different Aβ and p-tau protein residues, suggesting potential co-occurrence or co-expression of these proteins in AD pathogenesis [Figure 4].

Nodes:

- Colored nodes: Query proteins and first shell of interactors;

White nodes: Second shell of interactors;
Empty nodes: Proteins of unknown 3D structure;
Filled nodes: Proteins with known or predicted 3D structure.

Edges: Represent protein-protein associations, indicating proteins that jointly contribute to a shared function. The colors indicate different types of evidence for the association:

Blue: Known interactions from curated databases;

- Pink: Experimentally determined interactions;
- Green: Predicted interactions from gene neighborhood;
- Red: Predicted interactions from gene fusions;
- Dark blue: Predicted interactions from gene co-occurrence;
- Yellow: Interactions from text-mining;
- Black: Co-expression;

Light blue: Interactions from text-mining.

#### 3.3.2. Biomarkers Related to Abnormal Protein Accumulation in Non-Core AD Pathology

In addition to plasma biomarkers associated with core AD protein accumulation, other plasma-based molecules related to the abnormal protein accumulation of non-core AD pathogenesis have also been extensively studied.

##### Biomarkers of TAR DNA-Binding Protein (TDP-43) Accumulation

TDP-43 is a nuclear protein encoded by the TARDBP gene and is involved in various aspects of RNA processing, including transcription, splicing, and transport. It is a 43-kDa protein initially identified as a binding protein to the TAR (Trans-Activation Response) element of the HIV-1 virus [37,38]. TDP-43 pathology has also been observed in a subset of AD cases, particularly in association with hippocampal sclerosis, suggesting its involvement in a broader spectrum of neurodegenerative disorders [39]. Hippocampal sclerosis and TDP-43 are thought to be part of the later neuropathological changes in AD.

Given its role in neurodegenerative diseases, there is growing interest in using blood TDP-43 levels as a potential biomarker for conditions such as amyotrophic lateral sclerosis (ALS), frontotemporal dementia (FTD), and AD. Elevated levels of TDP-43 in blood, particularly in plasma or serum, have been observed in some studies of ALS and FTD patients, highlighting its potential as a non-invasive biomarker [40].

One of the significant challenges in measuring TDP-43 in blood is the sensitivity and specificity of the assays used. TDP-43 exists in multiple forms (e.g., full-length, truncated, phosphorylated), and distinguishing between these forms can be technically challenging [41,42]. Additionally, because TDP-43 is ubiquitously expressed in various tissues, its presence in blood may not always directly correlate with neurodegenerative disease [43].

TDP-43 and its phosphorylated form can be measured in platelet lysates. The antibody A-Phospho (S409/410-2) TDP-43 has been identified as a selective marker for AD, distinguishing AD from non-demented controls and amyotrophic lateral sclerosis (ALS) through platelet phospho-TDP-43 analysis. This AD-selective antibody may serve as a potential screening tool to enhance AD diagnosis, mainly when used alongside cognitive assessments [44]. Further studies are required to explore the profiles of phosphorylated TDP-43 in patient populations with MCI, mild dementia, and FTD.

##### BBBMs Associated with Synuclein Pathology

The brain afflicted by AD is neuropathologically characterized by the presence of extracellular amyloid-β (Aβ) plaques and neurofibrillary tangles composed of hyperphosphorylated tau proteins accumulating intraneuronally [45]. However, accumulating evidence suggests that the presynaptic protein α-synuclein (α-synuclein)—traditionally associated with Parkinson’s disease, Lewy body dementia, and multiple system atrophy—is also involved in the pathophysiology of AD [46]. Lewy-related pathology, primarily composed of α-synuclein, is present in a majority of autopsied AD brains, and higher levels of α-synuclein in the CSF of patients with MCI and AD have been linked to cognitive decline [45]. Recent studies suggest that asymptomatic accumulation of Aβ plaques is associated with elevated CSF α-synuclein levels in individuals at risk for sporadic AD and those with autosomal dominant AD [45]. Experimental evidence has further linked α-synuclein to tau hyperphosphorylation and the pathological actions of Aβ and Apolipoprotein E ε4, the latter being a major genetic risk factor for both AD and Lewy body dementia [47].

Therefore, the measurement of α-synuclein in body fluids, particularly blood, is crucial for detecting early AD pathology. However, a significant challenge arises from the presence of non-neurological sources of α-synuclein, such as red blood cells (RBCs), which contain it in abundance. Interestingly, 99% of total blood α-synuclein is found within blood components, with the majority residing in RBCs [48]. Due to their abundance and fragility, lysed RBCs can release α-synuclein into various fluid compartments of the body, including blood (where they typically remain) and CSF (where they can inadvertently enter), potentially leading to elevated α-synuclein levels in plasma or CSF [48].

Studies have shown that while total α-synuclein levels in the blood may not differ significantly between patients with neurodegenerative diseases and healthy controls, the levels of oligomeric or phosphorylated forms of α-synuclein might be more closely associated with disease states [48]. Therefore, assays with high specificity, capable of detecting even subtle variations in blood α-synuclein and effectively differentiating between its various forms, are critically needed. Interestingly, in a study, it was observed that the reduction of α-synuclein in the CSF of patients with Lewy body dementia was more pronounced than in AD patients or healthy controls, indicating a more significant accumulation of α-synuclein in the brain tissue of Lewy body dementia patients. In contrast, in AD, the decrease in CSF α-synuclein levels was not as significant and was comparable to that of healthy adults, suggesting different patterns of α-synuclein accumulation between these neurodegenerative conditions [48,49].

Regarding blood levels, a study conducted by Daniele et al. [50] demonstrated a significant difference in the levels of α-synuclein/tau and α-synuclein/Aβ42 heterodimers in the centrifuged RBCs of healthy controls compared to those with AD. Another study by Laske et al. [51] found that serum α-synuclein levels in AD and healthy controls were not significantly different, but both differed notably from the serum α-synuclein levels in LBD patients. As such, the correlation between serum α-synuclein levels in healthy controls and AD remains inconclusive and has yet to be adequately established [51].

Several studies suggest that Aβ42, tau, and α-synuclein interact in vivo to promote the aggregation and accumulation of each other, thereby accelerating cognitive dysfunction [52]. Interestingly, their expression levels and aggregation processes are not confined to the brain but also reach peripheral tissues via the bloodstream, suggesting the possibility that AD may be part of a systemic disease process [53].

##### Serum Dickkopf-1(DKK1) as Candidate BBBM in AD

DKK1 is a critical member of the DKK protein family and functions as a secretory glycoprotein with a significant role in determining cell fate in vertebrates. Recently, it has been implicated in both neurodegeneration and regeneration, with its role in AD becoming a focal point of research [54]. As an endogenous indirect inhibitor of the WNT/beta-catenin pathway, which plays an essential role in embryogenesis and adult homeostasis, DKK1 has been associated with cognitive decline in AD due to its dysregulation in both familial (early-onset) and sporadic (late-onset) AD cases [55]. Studies have shown that DKK1 expression increases significantly in the CSF, plasma, and brain tissue of AD patients and AD transgenic mice [54].

Research by Caricasole et al. [55] suggested that DKK1 induction initiates the pathological cascade of Aβ and enhances Tau phosphorylation. Other studies indicate that DKK1 inhibits endogenous WNT ligands, which are critical for synaptic maintenance [56]. Notably, the knockdown of DKK1 expression using siRNA in the hippocampus promotes hippocampal neuron regeneration and enhances both spatial working memory and memory consolidation, reversing age-related memory impairment [57].

Elevated serum concentrations of DKK1 enable it to cross the blood-brain barrier, potentially accelerating AD progression and making it a promising therapeutic target for AD treatment. Inhibiting DKK1 has been shown to improve spatial memory in animal models [58], with electrophysiological studies supporting DKK1’s role in **long-term potentiation [58].

Studies have reported that elevated serum levels of DKK1 in AD patients correlate with disease severity, particularly in terms of cognitive decline and synaptic loss. Elevated DKK1 in the blood can help identify individuals at risk of developing AD before clinical symptoms emerge [59]. Moreover, tracking serum DKK1 trends could provide insights into treatment prognosis and disease progression. Given its specific association with the Wnt signaling pathway and Aβ pathology, serum DKK1 levels may also help differentiate AD from other neurodegenerative conditions that do not involve this pathway disruption.

##### Plasma Visinin-like Protein-1 (VILIP-1)

VILIP-1 is an emerging biomarker that reflects various aspects of the heterogeneous pathophysiology of AD. VILIP-1 is a calcium-binding protein from the neuronal calcium sensor family, expressed in neuronal perikarya, dendrites, and some axons, playing a role in neuronal growth, survival, and synaptic plasticity [60]. In AD, disturbances in calcium homeostasis, followed by neuronal degeneration, lead to the release of VILIP-1 into the extracellular space.

A case-control study by Halbgebauer et al. [61] found that the SIMOA assay of CSF VILIP-1 and serum VILIP-1 could be highly sensitive and reliable for diagnosing AD. Their study reported a significant increase in CSF VILIP-1 levels in AD patients compared to control groups, as well as in patients with Parkinson’s disease, behavioral variant frontotemporal dementia, ALS, and Lewy body dementia. However, while serum VILIP-1 levels were elevated in AD patients compared to controls, there was no significant difference in concentrations between AD-MCI patients and other neurodegenerative groups [62].

These findings suggest that serum VILIP-1 alone may not be a reliable biomarker for early AD diagnosis. However, monitoring the CSF VILIP-1-to-serum VILIP-1 ratio could prove valuable for diagnostic purposes and understanding disease progression.

Table 1 provides a summary of various BBBMs related to abnormal protein accumulation in non-core AD pathology. Based on our STRING network model, interactions between TDP-43, α-synuclein, DKK1, and VLLIP-1 might suggest the co-occurrence or co-expression of these proteins in AD pathogenesis [Figure 4].

### 3.4. BBBMs of Neuronal and Synaptic Injury

In 2018, the NIA-AA guidelines incorporated neurodegeneration as the third biomarker (N) to define AD pathology. In their 2024 revised update, newly developed BBBMs for A, T, and N have been included [9]. Additionally, as previously mentioned, the group of biomarkers representing neuronal injury, dysfunction, or neuropil degeneration has been placed under the broader category of “Biomarkers of non-specific processes involved in AD pathophysiology.”

However, neurodegeneration or neuronal dysfunction alone may not be sufficient as a diagnostic marker, as its dynamic changes are more predictive of AD progression rather than initial diagnosis. In this context, biomarkers of synaptic dysfunction resulting from the loss of synaptic plasticity and integrity reflect the very early pathological changes in AD. Synaptic dysfunction is directly triggered by Aβ and tau pathology and indirectly by the consequences of neuroinflammatory responses.

#### 3.4.1. Plasma Neurofilaments as AD Diagnostic and Disease Progression Biomarkers

Neurofilaments are among the primary proteins expressed within neuronal cells, located in the axons, and play a critical role in maintaining the structural integrity and conduction velocity of nerve impulses, thus preserving the axonal caliber [63]. Degeneration of large-caliber axons is a hallmark of AD neurodegeneration.

Following neuroaxonal injury, there is a surge in neurofilament proteins in both blood and CSF. Recent breakthroughs have demonstrated the potential of plasma NfL in monitoring various aspects of neurodegeneration, including glucose metabolism, cognitive function, structural brain imaging, and future brain atrophy [64]. In a study by Mattsson et al. [65], patients with MCI, AD dementia, and those in the preclinical and prodromal stages of AD exhibited significantly higher baseline plasma NfL levels compared to controls. This study reinforces the potential of longitudinal tracking of NfL as a marker of neurodegeneration across various clinical stages of AD, including the preclinical phase.

Recent advancements have facilitated the measurement of neurofilament levels in blood samples, providing an alternative to the traditional approach of quantifying NfL in CSF. The development of 3rd/4th generation ELISA and the more sensitive electrochemiluminescence assay technology has revolutionized this process [66] have revolutionized this process. Interestingly, Gou et al. [19] found that plasma NfL is not significantly elevated until the MCI+ stage. Plasma NfL levels were notably higher in the MCI+ group compared to the cognitively unimpaired A-T- and cognitively unimpaired A+T- groups. A similar trend was observed in the AD dementia group, with plasma NfL levels elevated relative to the cognitively unimpaired A+T+, cognitively unimpaired A+T-, and MCI+ groups. Furthermore, SIMOA has enabled the detection of even slight disease-induced changes, including in healthy individuals [66]. NfL also shows promise as a treatment response biomarker for protopathic lesion-induced neurodegeneration [67].

#### 3.4.2. BBBMs Related to Pre-Synaptic Dysfunction

Synaptosome-associated protein 25 (SNAP-25) is a crucial protein located primarily in presynaptic vesicles and is linked to synaptic degradation. Studies have demonstrated an increasing trend in CSF SNAP-25 levels in the AD population, while a decreasing index in the cerebral cortex indicates the extent of synaptic dysfunction [68]. Notably, CSF SNAP-25 can differentiate AD from Parkinson’s disease and ALS, with elevated concentrations also found in Creutzfeldt–Jakob disease, highlighting its potential to distinguish between various neurodegenerative diseases [69]. In contrast, few studies have explored the association between plasma SNAP-25 and AD progression. Interestingly, a study by Agliardi et al. [70] revealed a decreasing trend in neuron-derived exosomes containing SNAP-25 in plasma, which correlated with cognitive status as measured by the Mini-Mental State Examination (MMSE).

Neuronal pentraxin 2 (NPTX-2), a protein associated with inhibitory circuit dysfunction, has shown promise as a biomarker of synaptic dysfunction. A longitudinal study by Libiger et al. [71] on CSF proteomics found a correlation between changes in NPTX-2 levels and the rate of cognitive decline. However, the role of NPTX-2 in AD remains unclear. Recent research has indicated a reduction in NPTX-2 levels in the plasma neuron-derived exosomes of AD patients, suggesting that this alteration could be detectable a decade before the onset of AD-associated dementia, making NPTX-2 a potential biomarker for early detection of AD [72].

Another potential biomarker of pre-synaptic dysfunction is growth-associated protein (GAP-43), which shows an increasing trend in CSF during AD dementia and correlates with Aβ burden and neurofibrillary tangle formation in regions such as the hippocampus, amygdala, and cerebral cortex [73]. While the association of plasma GAP-43 with AD remains uncertain, a recent study by Jia et al. [74] has highlighted the predictive potential of neuro-exosomal synaptic proteins, including GAP-43, neurogranin, SNAP-25, and synaptotagmin 1, which were shown to predict the development of AD 5 to 7 years before cognitive impairment becomes apparent [74].

#### 3.4.3. BBBMs Related to Post-Synaptic Protein Dysfunction

Neurogranin (NG), a 78 amino acid-long post-synaptic protein, is linked to synaptic dysfunction and neuronal injury [75]. Previous studies have highlighted the critical role of NG in maintaining synaptic plasticity, long-term potentiation, and long-term depression [76]. Elevated levels of CSF NG have been positively correlated with brain Aβ burden and tau pathology, with a specific fragment, NG 48–76, significantly increasing during the neurodegenerative process [77]. However, CSF NG lacks specificity for AD-related pathological changes, and plasma NG has not demonstrated any significant associative trend between AD and healthy controls [78]. Interestingly, a decreasing trend in plasma neuron-derived exosomes containing NG has been shown to positively correlate with cognitive decline, suggesting its potential as a marker for disease progression [79].

Table 1 illustrates different neuronal and synaptic injury-related BBBMs associated with AD pathology. Based on our STRING network model, interactions among proteins related to synaptic and neuronal injury may suggest the co-occurrence or co-expression of these proteins in AD pathogenesis [Figure 4].

### 3.5. Blood-Based AD-Related Biomarkers Associated with Vascular Pathology

The relationship between vascular pathology and BBBMs linked to AD offers crucial insights into the disease processes, particularly highlighting the interplay between neurodegeneration and vascular dysfunction.

#### 3.5.1. Fms-like Tyrosine Kinase-1 (Flt-1) in AD-Related Vascular Changes

Flt-1, also known as vascular endothelial growth factor receptor 1, plays a crucial role in AD. It serves as a receptor for vascular endothelial growth factors and is essential for regulating inflammation, vascular permeability, and angiogenesis. The disruption of the blood-brain barrier in AD is closely linked to Flt-1 dysregulation, which impairs angiogenesis and increases vascular permeability. This disruption facilitates the entry of harmful substances into the brain, promoting neuroinflammation and neurodegeneration [80,81].

A key pathological feature of AD is brain microvascular dysfunction, which has been associated with elevated Flt-1 levels. Recent research by Lau et al. [82] highlights the connection between enhanced angiogenesis, immune activation, and the endothelial overexpression of Flt-1 in AD. Measuring Flt-1 levels in the blood not only helps assess vascular involvement in AD but also presents a promising avenue for early detection of vascular changes associated with the disease, sparking further interest in this area of research.

#### 3.5.2. Role of Endothelin 1 (ET-1) in AD-Associated Vascular Pathology

Vascular endothelial cells primarily produce ET-1, a potent vasoconstrictor that plays a critical role in regulating vascular tone and blood flow. Elevated levels of ET-1 have been linked to vascular dysfunction and reduced cerebral blood flow in AD. ET-1 exacerbates neuronal damage through its association with tau pathology and Aβ deposition. Chronic vasoconstriction caused by ET-1 can lead to ischemia and hypoperfusion in the brain, further aggravating AD pathogenesis [83].

ET-1 levels may serve as indicators of vascular impairment in AD. A study by Palmer et al. [84] showed that ET-1 protein levels were significantly higher in AD tissue compared to controls, providing evidence of endothelin system overactivity in AD. This supports the idea that endothelin receptor antagonists may be valuable for treating AD.

#### 3.5.3. Alteration of Adrenomedullin (ADM) in AD

ADM regulates blood pressure, promotes vasodilation, and helps maintain the integrity of the endothelial barrier [85]. In AD, vascular dysregulation and endothelial dysfunction have been associated with altered levels of ADM. One of ADM’s neuroprotective effects is its ability to inhibit oxidative stress and inflammation, both of which are critical contributors to AD pathogenesis. Dysregulated ADM levels can lead to blood-brain barrier breakdown, potentially facilitating the entry of Aβ and other neurotoxic substances into the brain [86].

A study by Ferrero et al. [87] compared ADM levels in the cortex of AD patients and controls, revealing that ADM was significantly higher in the cortex of AD patients, further supporting its role in AD-related vascular pathology.

#### 3.5.4. Role of Atrial Natriuretic Peptide (ANP) in AD-Related Vascular Alterations

The cardiac ANP, which regulates salt homeostasis, fluid balance, and blood pressure, may offer the potential to treat AD. ANP plays a crucial role in cardiovascular homeostasis and exerts vasodilatory effects [88]. Given that individuals with AD often experience circulatory dysfunction, ANP levels may be influenced by the disease. It has been shown that ANP affects the risk of cerebral ischemia and modulates cerebrovascular tone [89]. Dysregulation of ANP signaling could contribute to reduced cerebral blood flow, potentially worsening the pathophysiology of AD.

Furthermore, ANP may play a role in facilitating the clearance of amyloid-beta (Aβ) from the brain [90]. Monitoring ANP levels in AD patients could provide insights into the cerebrovascular and cardiovascular aspects of the disease. A study by Mahinrad et al. [91] found an increased number of ANP receptors in AD brains compared to non-AD brains, suggesting that pathways related to ANP could present treatment opportunities for improving vascular function and mitigating AD progression.

#### 3.5.5. Vascular Immune Interaction and Monokine Induced by Gamma Interferon/C-X-C Motif Chemokine Ligand 9 (MIG/CXCL9) in AD

MIG/CXCL9, a chemokine, may hold significant diagnostic value in AD. Its primary function is to attract immune cells, especially T lymphocytes, to areas of inflammation [92]. It is involved in immunological surveillance and inflammatory reactions and is produced in response to interferon-gamma [93]. Elevated MIG/CXCL9 levels are linked to neuroinflammation and the migration of immune cells to the brain in AD. This chemokine aggravates amyloid pathology and neuronal impairment by contributing to the chronic inflammatory state seen in AD [94]. The blood concentration of MIG/CXCL9 may indicate ongoing neuroinflammatory processes and how the vascular and immune systems interact in AD. MIG/CXCL9 may serve as a biomarker for vascular-immune interactions and neuroinflammation in AD. Modulating MIG/CXCL9 or its signaling pathways could potentially offer therapeutic benefits in AD management.

#### 3.5.6. Role of Heart-Type Fatty Acid-Binding Protein (H-FABP) in AD-Related Vascular Pathology

AD can arise and evolve as a result of chronic vascular disease and impaired cerebral blood flow regulation. H-FABP plays a crucial role in fatty acid metabolism and lipid transport. Elevated levels of H-FABP indicate oxidative damage and systemic inflammation, potentially reflecting underlying vascular pathology. H-FABP levels are elevated in the CSF of patients with various neurodegenerative diseases, including AD, Parkinson’s disease with dementia, Lewy body dementia, vascular dementia, and Creutzfeldt–Jakob disease [95].

Several studies have demonstrated a positive correlation between H-FABP levels and its utility as a diagnostic and prognostic factor in AD. Desikan et al. [96] investigated H-FABP’s role in the earliest stages of AD, revealing that H-FABP levels were associated with atrophy in the entorhinal cortex and other brain regions particularly vulnerable to AD. Their study found that H-FABP levels correlated with p-tau and various apolipoproteins, including Apolipoprotein E and ApoCIII, suggesting a strong link between neuronal lipid biology and neurodegeneration [96].

Significantly, H-FABP was also associated with increased Aβ aggregation, highlighting the potential role of phospholipids, cholesterol, and protein transporters in Aβ dyshomeostasis [96]. These findings underscore the complex interplay between lipid metabolism and AD pathogenesis, pointing to H-FABP as both a potential biomarker for early detection and a possible therapeutic target in AD.

#### 3.5.7. Alteration of Vascular Adhesion Molecule (AM) Expression and Endothelial Dysfunction in AD

Endothelial dysfunction has been linked to cerebrovascular disease, with elevated levels of adhesion molecules (AMs) associated with the presence or progression of small and large vessel disease and white matter hyperintensities [97,98]. Studies have implicated adhesive proteins in multiple pathological mechanisms of MCI and AD, including amyloid plaque degradation, diffusion, and inflammation [99].

Soluble vascular cell adhesion molecule-1 (sVCAM-1), as opposed to soluble intercellular adhesion molecule-1 (sICAM-1), is more strongly associated with the atherosclerotic load as determined by angiography or echocardiography [100]. Thus, elevated sVCAM-1 levels might indicate the burden of atherosclerosis in AD and vascular dementia. Free β-amyloid inhibits endothelial nitric oxide synthase activity, causing endothelial dysfunction and increasing AM expression [101].

Several investigations measuring CSF and blood levels of intercellular adhesion molecule-1 (ICAM-1), VCAM-1, and interleukin-15 in AD have produced conflicting results, mainly due to differences in sample size, the cognitive status of controls, and the presence of confounding factors [102]. A study by Zuliani et al. [103] concluded that sVCAM-1 was elevated in vascular dementia and late-onset AD (without cerebrovascular disease), with no significant changes in E-selectin levels. Similarly, Drake et al. [104] demonstrated a positive association between sVCAM-1 and cognitive decline in AD but no correlation with ICAM-1 and E-selectin levels. A study by Chen et al. [99] identified VCAM-1 and activated leukocyte cell adhesion molecule levels (ALCAM) as strong predictors of AD, showing a significant correlation between age and the severity of cognitive decline, with no significant changes in ICAM levels. In contrast, a study by Janelidze et al. [102] reported a substantial increase in ICAM-1, VCAM-1, YKL-40, interleukin-15, and Flt-1 in AD’s preclinical and prodromal stages associated with cognitive decline and increased risk of subsequent AD development.

Table 1 summarizes various vascular pathology-related BBBMs of AD. Based on our STRING network model, interactions between proteins associated with AD-related vasculopathy might suggest the co-occurrence or co-expression of these proteins in AD pathogenesis. [Figure 4].

### 3.6. BBBMs Associated with Oxidative Stress and Bioenergetics

#### 3.6.1. BBBMs Related to Oxidative Stress

Oxidative stress can accompany AD and mild cognitive impairment (MCI)-related pathological changes and is considered a crucial upstream factor in disease progression. The products of free radical damage, such as aldehydes and lipid hydroperoxides, can readily diffuse into the peripheral circulation. Studies have revealed that blood-brain barrier permeability and integrity are significantly affected in both AD and vascular dementia, and products of oxidative stress represent potential BBBMs for AD diagnosis [105,106]. However, oxidative stress markers in the blood in AD are inconsistent as they can be influenced by underlying co-morbidities such as diabetes, metabolic syndrome, or cardiovascular diseases.

Notably, AD-related oxidative stress is due to Aβ misfolding, which activates resting microglia. The NADPH oxidase inside the microglia is activated, leading to free radical generation in AD patients [107]. Additionally, the Aβ peptide is an essential source of free radicals in AD, and it has been found that Aβ directly produces free radicals, for which methionine at the 35th position is responsible [108]. Moreover, Aβ binds with redox-active metals, which function as a catalytic factor for free radical production. In this context, Fe^2^⁺ concentration is increased in the AD brain. Furthermore, the oxidative stress-related burden precedes the formation of senile plaques and tangles [109,110].

Metabolic products secondary to lipid peroxidation accumulate in neurons without AD-related pathological changes, and these brain-formed intermediates may easily traverse the blood-brain barrier, given their small size and lipophilic nature [111]. Several studies have highlighted the importance of malondialdehyde, primarily arising from polyunsaturated fatty acid, and 4-hydroxynonenal, another essential product of linoleic and arachidonic acid peroxidation, as potential BBBMs of brain oxidative stress in AD [112]. In plasma, both malondialdehyde and 4-hydroxynonenal levels are increased in MCI-AD compared to controls [113,114].

Importantly, isoprostanes represent the best available biomarkers of lipid peroxidation today. Several studies have shown that increased F2-isoprostanes in body fluids, including plasma, CSF, and urine, are potential markers of oxidative stress during the MCI phase of AD [115]. Furthermore, their concentration correlates with the disease continuum, from subjective cognitive decline to MCI to AD. However, within MCI and AD groups, F2-isoprostanes did not correlate with memory impairment duration or cognitive test scores [116].

Interestingly, fibroblasts and lymphoblasts from patients with familial AD, in contrast to sporadic AD, carry APP and presenilin-1 gene mutations and show an increase in malondialdehyde and 4-hydroxynonenal levels [117].

Free radicals may further impair essential proteins’ structural and functional properties directly or secondarily by attacking them as end-products of lipid peroxidation [118]. The reaction of various reactive oxygen species and reactive nitrogen species can lead to the formation of 3-nitrotyrosine and dinitrotyrosine, and their concentration is increased in the CSF and plasma of AD individuals [119]. Additionally, MMSE scores correlated negatively with 3-nitrotyrosine concentration in CSF, and the total protein nitration measure in brain samples differed significantly in MCI compared to healthy controls in the inferior parietal lobule and hippocampus [119,120].

Studies using a plasma proteomic approach have revealed that specific oxidation products in AD, identified as isoforms of human transferrin, hemopexin, alpha-1-antitrypsin, and fibrinogen gamma-chain precursor proteins, are significantly increased [121]. Furthermore, elevated levels of carbonyl proteins and tyrosine in immunoglobulin G have also been documented in AD. However, only one study reported increased levels of 3-nitrotyrosine and dinitrotyrosine in the plasma of AD patients [122].

Notably, oxidative damage to DNA or RNA, primarily through the chemical modification of DNA bases or deoxyribose, has been measured in individuals with AD. One of the most significant findings in assessing DNA oxidative damage in AD is the elevated levels of 8-hydroxyguanosine, which are markedly higher in the lymphocytes of AD patients compared to controls [123]. Other studies have reported increased levels of oxidized pyrimidines and purines in the peripheral blood of AD patients relative to age-matched controls. This oxidative damage to DNA in plasma occurs much earlier in the pathogenesis of AD [124].

Additionally, studies on antioxidant levels in the blood have shown a reduction due to increased oxidative stress in the early stages of AD. Several studies have demonstrated decreased plasma levels of vitamins E, C, and A in AD patients under normal dietary conditions without supplementation [125]. However, some studies have not found significant differences in plasma antioxidant levels between AD patients and controls. Overall, the total antioxidant capacity of plasma is significantly reduced in AD patients and is negatively correlated with disease duration [126].

#### 3.6.2. Blood-Based Bioenergetic Profiling

Blood-based bioenergetic profiling has emerged as a reliable and minimally invasive method for assessing mitochondrial function. Circulating blood cells, including platelets and peripheral blood mononuclear cells, exhibit high rates of electron transport chain activity and metabolic flexibility. According to the mitochondrial cascade hypothesis, mitochondrial dysfunction, particularly in the form of reduced mitochondrial respiration, is one of the earliest hallmarks of AD and a key contributing factor to the formation of senile plaques and neurofibrillary tangles, particularly in sporadic or late-onset AD individuals [127].

In parallel, disruptions in mitochondrial quality control processes—such as fusion, fission, and autophagy—can trigger neurodegeneration. Mitochondrial DNA (mt-DNA) mutations have been correlated with an increased risk of cognitive decline and AD pathogenesis [128]. Additionally, Aβ deposition has been associated with impaired mitochondrial bioenergetics, including electron transport chain uncoupling, reduced ATP production, and increased reactive oxygen species generation, further contributing to AD pathology [129].

Notably, peripheral blood mononuclear cells from early AD patients revealed decreased expression of mitochondrial respiratory complex I-V genes and mitochondrial ribosomal complex subunits compared to the control group [130]. These changes lead to accelerated mitochondrial dysfunction and enhanced oxidative damage. Studies have shown that peripheral blood mononuclear cells from sporadic AD patients had decreased basal oxygen consumption rate and proton leak without changing maximum respiratory capacity compared to age-matched controls [131]. Concurrently, a reduction in basal oxygen consumption rate and maximum respiratory capacity was evident in lymphocyte mitochondria [132]. Interestingly, platelets have also been found to have abnormalities in the electron transport chain of AD patients and are considered an emerging biomarker in peripheral blood in AD patients [133]. Several studies have reported decreased activity and expression of the complex-IV enzyme and its subunits in the platelets of AD individuals [130].

Additionally, fibroblasts from both sporadic and familial AD cases have revealed functional abnormalities and an increase in their numbers in peripheral circulation. The electron transport chain function in fibroblasts from AD patients reflects higher variability than in other blood cells [134]. Studies have also reported impaired glucose uptake by the fibroblasts from AD individuals [135]. However, it remains unclear whether the functional and structural changes of the electron transport chain seen in AD individuals are due to primary mitochondrial changes (mitochondrial hypothesis of AD) or Aβ deposition-mediated alterations.

Mitochondrial dynamics have also been found to be significantly impaired in AD individuals and can be a potential approach to studying these changes as biomarkers of early AD dementia. In this context, the formation of a complex formed by S-nitrosothiol and dynamin-related protein 1 (Drp1), i.e., SNO-Drp1, can result in increased mitochondrial fission, loss of synapses, and neuronal damage in mouse models and primary neuronal cultures as well as in post-mortem tissue. SNO-Drp1 is increased in peripheral blood lymphocytes in AD patients, and preventing nitrosylation has been found to reduce neuronal loss [135]. There are also contradictory findings that SNO-Drp1 does not differ significantly in AD compared to controls [136].

The current literature has also suggested the importance of studying mitochondrial calcium signaling as a promising biomarker. Interestingly, AD’s abnormal mitochondrial Ca^2^⁺ concentration and signaling are directly associated with Aβ and tau protein or with the mutated presenilin 1 and presenilin 2 genes in familial AD [137].

Recent studies on blood-based indices of mitochondrial DNA (mt-DNA) copy number and cell-free mtDNA have revealed that mt-DNA copy number was significantly associated with cognitive impairment and cell-free mtDNA was also found to be higher in these cases when compared to controls [138]. Hence, cellular mt-DNA copy number can be used as a potential biomarker of mitochondrial biogenesis and cellular energetics to reflect mitochondrial health in AD. In this context, a study by Reid et al. [139] has revealed that assessment of 8-oxo-7,8-dihydro guanine (8oxoG) somatic single nucleotide variants (sSNVs) can serve as a better mitochondrial dysfunction-related biomarker. In contrast, due to its inflammatory endophenotype, the circulating cell-free mtDNA 8oxoG variant can be used as an improved biomarker [140].

The most critical challenge of using mitochondrial function as a biomarker is the variability of the measurement methods used. Functional assessment of mitochondria requires several weeks of processing biological samples from patients. This would be expensive and time-consuming, making the development of a practical, widely available mitochondria-based biomarker difficult. Moreover, only a handful of studies have investigated mitochondrial abnormalities in AD by recruiting large patient cohorts. Therefore, further investigations on a larger scale are needed to identify more precise and accurate mitochondrial function-related abnormalities that will be considered standard biomarkers in the future.

In Table 1, the oxidative stress and bioenergetic dysfunction-related biomarkers in AD pathology are highlighted. The STRING network model indicates interactions between proteins associated with oxidative stress and mitochondrial dynamics, suggesting their co-expression or co-occurrence in AD, as depicted in Figure 4.

### 3.7. BBBMs of Neuroinflammation and Immune Dysregulation

One of the most relevant factors in the pathogenesis of AD is chronic neuroinflammation and the involvement of microglia in this process [141]. These inflammatory markers can serve as potential supplements to the AD diagnostic panel. Most of these inflammatory biomarkers can be analyzed from blood or its derivatives, with their concentrations quantifiable by ELISA or other immunoassays, such as electrochemiluminescence immunoassay and the Mesoscale Discovery Immunoassay V-PLEX.

AD is characterized by prominent astrogliosis, often seen surrounding amyloid plaques, with activated astrocyte processes participating in neuritic plaque formation [142]. The intermediate filament glial fibrillary acidic protein (GFAP) is highly upregulated in reactive astrocytes. These reactive astrocytes contribute to neuroinflammation by releasing pro-inflammatory cytokines and chemokines, which in turn exacerbate the progression of AD [143]. Preliminary data on GFAP suggest that the plasma biomarker performs better than its CSF counterpart in identifying AD pathology [144]. Several studies have shown marked elevations of GFAP in AD and MCI without significant changes in FTD and progressive supranuclear palsy. This elevation is associated with longitudinal declines in revised Addenbrooke’s Cognitive Examination scores in MCI and AD, adding prognostic value [145].

Benedet et al. [146] conducted a comprehensive study measuring GFAP levels across the entire AD continuum. Their findings revealed that plasma GFAP levels, in contrast to CSF GFAP levels, were elevated in individuals with preclinical AD, significantly increasing as the disease progressed to symptomatic stages. Notably, the study also found a positive correlation between plasma GFAP levels and Aβ pathology [146]. It is noteworthy that Gou et al. [19], as mentioned earlier, revealed dynamic trends through the AD continuum [Figure 1B], where plasma GFAP levels were significantly increased in cognitively unimpaired Aβ-positive tau-negative (A+T-) compared to cognitively unimpaired Aβ-negative tau-negative (A-T-) groups. The same trend was observed in cognitively unimpaired Aβ-positive tau-positive (A+T+) compared to cognitively unimpaired A-T- groups, as well as in the AD dementia group relative to cognitively unimpaired A+T+ and cognitively unimpaired A+T- and MCI+ groups.

Several pro-inflammatory cytokines, including interleukin-1 alpha, interleukin-1 beta, interleukin-6, and chemokines such as monocyte chemoattractant protein-1, are altered in the serum and CSF of AD individuals compared to controls [147]. However, such alterations may not be directly associated with AD and can relate to aging and other systemic diseases. White blood cell CX3CL1, also called fractalkine, is significantly elevated in the plasma of MCI and AD patients [148]. C-C motif chemokine ligand 23 plasma concentration has also been found to have predictive value regarding MCI-to-AD progression. C-C chemokine ligands, or regulated upon activation, normal T cell expressed and secreted (RANTES), have elevated plasma concentrations in AD, correlating with the neuroinflammatory burden [149].

Progranulin, a growth factor expressed in neurons and microglia, has been associated with neuroinflammatory modulation, such as microgliosis and astrogliosis. Studies have revealed that increased levels of the progranulin-expressing gene are found in the blood of MCI and AD patients [150,151]. Interestingly, YKL-40, a chitinase-3-like protein (encoded by the CHI3L1 gene), is increasingly expressed in astrocytes during neuroinflammatory changes. A longitudinal study has revealed that plasma YKL-40 levels are negatively correlated with cognitively healthy individuals at risk of developing AD and show a positive correlation with the results of the sensitive Free and Cued Selective Reminding Test [152].

Additionally, interleukin-33 and the soluble form of its receptor (soluble suppression of tumorigenicity 2) are associated with neuroinflammation. Interestingly, in AD, interleukin-33 plays a protective role and is found to be downregulated in the brain tissues of MCI and AD individuals. However, its plasma concentration is higher in MCI and AD than in controls. Hence, a higher plasma concentration of interleukin-33 is associated with better cognitive function [153]. The question remains: why is this cytokine elevated in AD and MCI? A possible answer could be that AD patients have a higher concentration of soluble suppression of tumorigenicity 2, which attenuates the effective concentration of interleukin-33, contributing to cognitive function in the AD disease continuum [154].

Recently, increased levels of triggering receptor expressed on myeloid cells 2 (TREM2) messenger RNA levels in peripheral mononuclear cells have been found to distinguish between amnesic MCI, AD, and healthy control individuals, depending on the Apolipoprotein E genotype [155]. A study by Hu et al. [156] found that TREM2 expression in monocytes is consistent with RNA-based observations in circulating monocytes. Like TREM2, TREM1 has also been identified as a potential biomarker [157]. Interestingly, the soluble form of secreted TREM2 concentration is lower in plasma and associated with Aβ accumulation and CSF p-Tau levels in AD [158]. However, such alterations in secreted TREM2 have also been found in vascular dementia, raising questions about the specificity of this biomarker.

The various blood-based biomarkers related to neuroinflammation and immune dysregulation in AD pathology are detailed in Table 1. Our STRING network model indicates potential interactions, suggesting co-expression or co-occurrence of these proteins in AD pathogenesis, as depicted in Figure 4.

### 3.8. Blood-Based Epigenetic Biomarkers Related to Early Detection and Prognosis of AD

Epigenetics has proven to be a valuable tool for gaining a deeper understanding of the pathogenesis of AD. In the quest for more relevant blood biomarkers for AD, the role of epigenetic mechanisms—those that mediate the interaction between the genome and the environment—is becoming increasingly significant. These mechanisms are emerging as key contributors to the pathogenesis of AD.

#### 3.8.1. DNA Methylation-Based Markers

DNA methylation, an epigenetic marker influenced by both genetic inheritance and environmental factors, demonstrates significant potential for predicting AD. Blood methylation patterns may offer valuable insights into the biological mechanisms underlying AD pathogenesis. Specific methylation changes have been linked to neurodegenerative processes, suggesting that these epigenetic alterations could serve as early indicators of AD risk. The ability to identify these methylation changes years before the onset of clinical symptoms underscores DNA methylation as a promising approach for early diagnosis and intervention in AD. This is particularly relevant given that the pathological characteristics of AD can manifest long before cognitive decline becomes noticeable [159].

Recent studies have explored the role of DNA methylation in creating “epigenetic clocks”, which link biological age to disease mechanisms. Changes in blood DNA methylation have been associated with CSF biomarkers of AD, including Aβ and tau proteins. A comprehensive analysis involving over 111,000 AD cases and nearly 678,000 controls identified 1168 cytosine-phosphate-guanine sites significantly associated with AD risk. Among these, 52 cytosine-phosphate-guanine sites related to 32 genes demonstrated consistent associations with AD risk. Notably, this analysis revealed potential risk genes, such as CNIH4 and THUMPD3, which were not previously recognized in AD etiology [160].

#### 3.8.2. Potential Blood-Based microRNA Biomarkers for AD

MicroRNAs play a complex role in the development of AD, influencing various aspects of the condition, including Aβ metabolism, tau phosphorylation, neuroinflammation, and synaptic function.

A comprehensive literature review identified 137 microRNAs altered in the blood of AD patients, with 36 confirmed in at least one independent study [161]. The most frequently reported microRNAs include hsa-miR-146a, hsa-miR-125b, and hsa-miR-135a, which have been consistently found to be altered in blood, CSF, and brain tissue of individuals with AD [161]. One study identified a three-microRNA signature (miR-92a-3p, miR-486-5p, miR-29a-3p) that may differentiate between preclinical AD, MCI due to AD, and healthy controls [162]. Another study proposed a panel of 12 microRNAs for predicting AD progression in patients with MCI, with eight validated through quantitative polymerase chain reaction [163]. Dysregulated microRNAs in the blood of AD patients have been linked to various pathways associated with AD pathogenesis, including regulation of APP cleavage, expression of presenilin-1 and beta-site APP cleaving enzyme 1 (BACE1), oxidative stress, neuroinflammation, cell cycle regulation, synaptic transmission, cell signaling, and metabolism [162,164,165].

**Figure 5:**
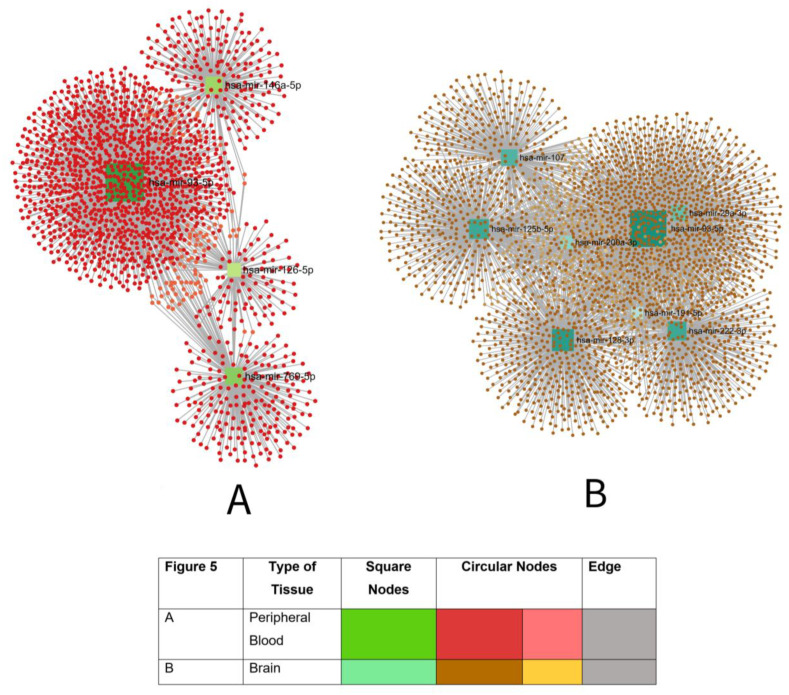
The figure illustrates the microRNA interaction networks for predicted microRNA markers of AD in peripheral blood and brain tissue. (**A**) Depicts the network for four predicted microRNA markers in peripheral blood. The network is characterized by a densely interconnected structure, with four prominent green square nodes representing the predicted microRNAs. These square nodes vary in size, indicating differences in the number of gene interactions each microRNA has. Surrounding these microRNAs are numerous circular nodes in shades of red, representing target genes. The darker red nodes signify genes without interactions with other genes, while lighter red nodes indicate genes interacting with multiple others. (**B**) Shows the network for eight predicted microRNA markers in brain tissue. This network appears more dispersed compared to A, with eight green square nodes of varying sizes representing the predicted microRNAs. The circular nodes in this network are in shades of brown, with darker brown indicating isolated genes and lighter brown showing genes with multiple interactions.

A comparative analysis of microRNA networks between peripheral blood and brain tissue [Table 2] revealed that miR-93-5p exhibits significant interactions with validated genes in both tissues [Figure 5]. This finding is clinically significant, as it could aid in early diagnosis of disease progression monitoring and enhance our understanding of AD pathophysiology. The analysis found that miR-93-5p interacts with the majority of genes in both blood and brain tissues. Its increased presence in these tissues among AD patients may have a detrimental effect by regulating multiple genes associated with various pathways.

These findings suggest that miR-93-5p could be a promising candidate for future blood-based biomarkers in AD, potentially facilitating early diagnosis and tracking disease progression.

In both networks, grey edges connect the nodes, representing microRNA-gene interactions or gene-gene interactions. The complexity and density of these connections highlight the intricate regulatory relationships in AD pathology.

The accompanying table clarifies the color coding, confirming that green square nodes represent predicted microRNAs, while the circular nodes’ colors (red for peripheral blood, brown for brain tissue) represent target genes, with intensity indicating the degree of gene interaction.

This visualization effectively demonstrates the complex interplay between microRNAs and their target genes in different tissues relevant to AD, providing insights into potential biomarkers and regulatory mechanisms involved in the disease.

#### 3.8.3. Potential Blood-Based Long Non-Coding RNA (lncRNA) Biomarkers for AD

Recent studies have identified multiple lncRNAs in blood that may serve as promising biomarkers for the detection and progression of AD. Plasma levels of NEAT1 and BC200 were significantly elevated in AD patients compared to healthy controls, effectively differentiating AD patients with high sensitivity and specificity. NEAT1 could also distinguish between MCI and advanced AD compared to controls. Furthermore, plasma levels of BC200 showed a positive correlation with patient age [166].

Plasma levels of BACE1 lncRNA were significantly elevated in AD patients compared to non-AD controls. Receiver operating characteristic (ROC) analysis demonstrated that BACE1 exhibited high specificity (88%) for AD [167]. Other lncRNAs, such as NDM29, FAS-AS1, and GAS5-AS1, were assessed but did not reveal significant differences between AD patients and controls [166]. In silico analysis of RNA-sequencing data identified 33 upregulated and 13 downregulated lncRNAs in AD patients compared to controls [167]. These studies indicate that plasma levels of NEAT1, BC200, and BACE1 lncRNAs are promising BBBMs that could assist in early AD detection and disease progression monitoring. However, more extensive validation studies are necessary to confirm their clinical applicability. Integrating these lncRNA biomarkers with other BBBMs, such as amyloid-beta and tau proteins, may further enhance diagnostic accuracy.

#### 3.8.4. Markers Related to Histone Modification and DNA Alteration

Histone modifications, including acetylation, methylation, and phosphorylation, are essential for regulating gene expression and have been shown to affect cognitive functions, learning, and memory. In AD, research suggests both losses and gains of specific histone marks, indicating a complex interaction of epigenetic changes contributing to disease progression and pathology [168,169]. Histone acetylation is linked to active gene expression, while methylation can activate or repress genes based on the specific context. In AD, changes in these modifications are associated with cognitive decline and neurodegeneration, underscoring their potential as biomarkers [168].

DNA methylation age, reflecting biological aging, has also been correlated with AD pathology, indicating that accelerated epigenetic aging may contribute to the disease [170,171]. Research has demonstrated that differential methylation occurs in genomic regions associated with AD susceptibility, suggesting that these changes may precede clinical symptoms and act as early disease indicators [168,169].

The dynamic nature of histone and DNA modifications creates opportunities to develop diagnostic and prognostic biomarkers for AD. These epigenetic markers could assist in early detection, tracking disease progression, and assessing treatment responses. Moreover, targeting these modifications with epigenetic drugs, such as histone deacetylase inhibitors, is being investigated as a therapeutic approach to alter disease outcomes [168,169,170,171].

#### 3.8.5. Circular RNA (circRNA)-Related Biomarkers

CircRNAs have been identified as potential biomarkers for AD, presenting an encouraging pathway for early diagnosis and differentiation from other forms of dementia. One study established a panel of six circRNAs that effectively distinguish AD patients from cognitively healthy individuals and those with different types of dementia, such as vascular dementia and Parkinson’s disease dementia. This panel was validated using three independent datasets, demonstrating its specificity and potential as a dependable biomarker for AD [172].

Distinct expression profiles of circRNAs in AD have been revealed, with certain circRNAs being either upregulated or downregulated in the blood of affected patients. For example, hsa_circ_0003391 has been identified as significantly downregulated in AD patients compared to those with other types of dementia, indicating its potential as a diagnostic marker [173].

The ability of circRNAs to traverse the blood-brain barrier, combined with their specific expression profiles in various tissues, enhances their potential as biomarkers. They could aid in creating non-invasive diagnostic tests for AD, enabling earlier intervention and improved disease management [174,175]. CircRNAs play a significant role in AD pathophysiology, functioning as ‘sponges’ for microRNAs and thereby influencing gene regulation and potentially contributing to disease progression. Their stability and abundance in the nervous system make them appealing candidates for biomarker development.

### 3.9. Plasma Exosome-Based AD-Related Biomarkers

Exosomes derived from CSF, blood, and neural cells have emerged as potential biomarkers for diagnosing AD. Proteomic analysis of CSF-derived exosomal vesicles (EV) revealed more than 400 unique proteins involved in AD pathogenesis [176]. Muraoka et al. [177] identified proteins such as HSPA1A, NPEPPS, and PTGFRN as essential for monitoring the progression of MCI to AD. T-tau and p-181-tau levels in CSF-derived EVs were higher in AD patients compared to healthy controls [178]. The encapsulating lipid bilayer of exosomes allows efficient crossing of the blood-brain barrier without losing biomarkers, reaching various biological fluids such as blood, urine, saliva, and synovial fluid [179].

Fiandaca et al. [180] demonstrated the predictive power of neuronal-derived exosomes in AD. Levels of p-tau proteins, specifically P-S396-tau and p-tau181, in neuronal-derived exosomes could predict sporadic AD development up to 10 years before clinical onset. The study also found elevated levels of several globin proteins in neuronal-derived exosomes of AD patients compared to controls [180]. In contrast, plasma neuronal-derived exosomes showed lower levels of presynaptic proteins (synaptotagmin, synaptophysin) and post-synaptic proteins (synaptopodin, neurogranin) [181].

Metabolism-based blood neuronal-derived exosomes include P-S312-IRS-1, which showed higher levels in AD than control subjects without changes in T-IRS-1. Down-regulation of P-panY-IRS-1 and N-(1-carboxymethyl)-L-lysine has been reported in AD patients compared to controls. Other neuronal-derived exosome biomarkers, such as Ser/Tyr phosphorylation of insulin receptor substrate 1, lysosomal enzymes, and ubiquitin, show marked differences between AD patients and healthy controls [182].

Astrocyte-derived exosomes have also emerged as potential biomarkers for AD diagnosis. Levels of complement proteins (C1q, C3b) and cytokines (interleukin 6, TNF-α, interleukin-1 beta) in astrocyte-derived exosomes significantly differed between AD individuals and controls. Elevated levels of C1q, C4b, factor D, fragments Bb, C5b, C3b, and C5b-C9 in plasma astrocyte-derived exosomes could serve as predictive biomarkers for MCI progression to AD [183].

Research indicates that microglia can phagocytose tau-containing neurons and synapses and transfer tau to other neurons via exosomes, establishing a connection between microglia, their derived exosomes, and tau pathology. Reducing microglial presence impedes tau propagation. Activated microglia can release exosomes containing inflammatory markers and pro-resolving genes, resulting in a more damaging pro-inflammatory state throughout the brain [180,184]. However, it has been reported that microglia and neighboring neurons could collaborate to clear Aβ peptides through exosomes. However, microglia and neighboring neurons may collaborate to clear Aβ peptides through exosomes.

A summary of various exosome-based BBB models related to AD is provided in Table 1.

## 4. Differential Expression of BBBMs in Hereditary Subtypes of AD and Non-Alzheimer’s Dementias

Unusual features in blood measures may indicate mutations in hereditary AD types, particularly those linked to early-onset AD associated with mutations in presenilin 1, presenilin 2, and APP genes [185]. While blood biomarkers for AD have conventionally focused on sporadic late-onset AD, recent developments have provided insights into familial variants of the disease.

Weston et al. [186] observed that individuals carrying presymptomatic mutations for familial AD forms exhibited elevated serum NfL levels compared to non-carriers. These carriers harbored pathogenic mutations in genes responsible for presenilin 1 and APP, indicating discernible neurodegeneration even during the presymptomatic phase [187]. A longitudinal study involving APPswe and APParc mouse models and presenilin 1 mutations in a Swedish autosomal dominant AD cohort revealed higher levels of plasma GFAP, p-tau181, and NfL in mutation carriers compared to controls. Approximately a decade before symptom onset, GFAP showed an initial increase, followed by p-tau181 and NfL. Further research is needed to substantiate these findings [188].

Genetic variations, primarily in presenilin 1, affect Aβ synthesis and γ-secretase activity, leading to autosomal dominant AD. These variations cause differences in biomarker levels, cognitive impairment, and age at symptom onset. Schultz et al. [189] investigated the impact of PSEN1 variants on γ-secretase activity and Aβ production in mutation carriers. Their findings suggested two critical implications: the potential of γ-secretase as a therapeutic target in AD and the usefulness of cell-based models in improving predictions of symptom onset. Notably, the study proposed that the varying effects of different PSEN1 variants on γ-secretase activity and Aβ production could account for some of the clinical heterogeneity observed in individuals with autosomal dominant AD. This research provides valuable insights into the molecular mechanisms underlying autosomal dominant AD and suggests potential avenues for personalized treatment approaches [189].

A recent study examined the relationship between soluble TREM2 and autosomal dominant AD progression. The findings suggest that TREM2 may play a role in Aβ plaque deposition and could have protective effects on cognitive decline. Soluble TREM2 could be an essential marker for planning therapeutic trials, and the development of TREM2-boosting treatments is ongoing [190]. Table 3 summarizes the differential expression of BBBMs between hereditary and sporadic AD.

Multiple studies have highlighted the significance of plasma-based biomarkers, particularly Aβ ratios, p-tau, t-tau, NfL, and GFAP, in advancing the diagnostic and prognostic framework for AD and potentially other types of dementia. The exceptional diagnostic accuracy and specificity of these biomarkers position them as viable alternatives to traditional CSF and imaging-based diagnostics for characterizing various forms of dementia [191,192,193,194]. Table 4 provides a summary of the differential expression of AD-related BBBM in comparison to FTD, LBD, and vascular dementia.

## 5. Current Challenges and Limitations in Incorporating AD-Associated BBBMs in Clinical Practice

### 5.1. Lack of Standard Cut-Off Points in Plasma-Based Assay Techniques and Optimal Study Design

Despite recent advancements in the identification and application of BBBMs for diagnosing AD, several challenges remain in refining assay techniques, conducting longitudinal validation, and using multi-modal approaches. A fundamental limitation is the lack of standardized cut-off points for plasma-based assays, complicating their clinical utility. Additionally, many observational studies and clinical trials suffer from poor cohort representation. Studies conducted in underrepresented populations often rely on convenience samples, introducing selection bias and limiting generalizability. Therefore, epidemiological diversification and the inclusion of real-world data in biomarker studies are crucial to establishing population-level validity.

Challenges also persist in standardizing biomarker assays across different populations and integrating these methods into routine clinical workflows. While non-invasive BBBMs show potential for early detection and monitoring of AD-related pathology, they do not yet provide definitive diagnostic accuracy [195]. Further validation of BBBM integration with current diagnostic practices is essential. Nevertheless, these biomarkers hold promise in enhancing clinical decision-making and guiding therapeutic interventions for AD.

### 5.2. Changes in AD-Related BBBMs across Various Chronic Conditions

Peripheral physiological and pathophysiological factors can influence blood-based AD biomarkers more readily than CSF-based markers, increasing the risk of false positives or negatives in diagnosing AD. For example, studies have shown that chronic kidney disease can elevate plasma levels of Aβ42, Aβ40, pTau181, pTau217, and NfL, likely due to impaired renal clearance, which raises the risk of falsely diagnosing AD in patients with chronic kidney disease [196,197].

Similarly, obesity and metabolic syndrome have been associated with lower plasma NfL levels, possibly due to greater blood volume in these individuals [198]. However, t-tau levels are positively correlated with body mass index, suggesting that metabolic factors may differentially affect various biomarkers [199].

Age is another critical factor influencing AD-related BBBMs. Research has demonstrated positive correlations between advancing age and biomarkers such as Aβ42, Aβ40, NfL, and t-tau. Interestingly, while these individual biomarkers tend to increase with age, the Aβ42/40 ratio typically decreases as age progresses [200]. Moreover, age-related changes in Aβ transport across the brain’s barriers complicate the interpretation of blood biomarker data. Peripheral amyloidosis, particularly in patients with insulin resistance or metabolic syndrome, may also affect plasma Aβ42 and Aβ40 levels, leading to potential misinterpretations of AD-related biomarkers [201].

Cholesterol levels have also been shown to influence amyloid-beta metabolism, with elevated cholesterol-altering APP processing and increasing Aβ production, which may skew blood-based measurements [202]. Additionally, some studies have observed gender differences in biomarker levels, such as higher t-tau levels in women and increased Aβ40 levels in men; however, these findings require further validation in larger cohorts [203].

Patients with cardiovascular disease, particularly those with chronic heart failure or on neprilysin inhibitors (e.g., sacubitril), may exhibit altered AD biomarker levels. NEP inhibitors impair Aβ degradation in both the brain and peripheral circulation, leading to reduced Aβ42/40 ratios and elevated plasma Aβ42 and Aβ40 levels [204].

Cancer, particularly glioblastoma and other tumors can also interfere with BBBMs. Tumor-associated neuroinflammation can elevate p-tau levels, which may not be related to AD pathology but instead to tumor-induced metabolic changes [205]. A recent review by Couch et al. [206] highlighted the role of EVs associated with L1CAM in neurodegenerative diseases like AD and various cancers. L1CAM, a marker of neuron-derived EVs, is upregulated in many cancer types, complicating the differentiation between AD-specific EV biomarkers and those associated with oncological conditions. Therefore, developing precise methods for characterizing the plasma EV population is crucial for evaluating biomarkers in overlapping diseases.

Controlling these factors during clinical evaluations and research is essential to improving the accuracy of BBBMs for diagnosing AD. Large-scale studies that account for variables such as cholesterol levels, tumor presence, kidney function, and other confounding factors will be essential in establishing reliable diagnostic cut-offs and reducing variability in biomarker measurements.

## 6. Future Directions and Conclusions

Recent advancements in BBBM have significantly transformed the approach to AD diagnosis. Non-invasive tests, such as those utilizing immunoprecipitation and liquid chromatography-mass spectrometry, have enhanced the detection of pathological changes in AD, offering an alternative to more invasive CSF analysis and costly PET scans. Plasma biomarkers, including normalized (APP)669–711/Aβ1–42 and Aβ1–40/Aβ1–42, have shown high accuracy in reflecting CSF levels, further establishing their diagnostic utility. Combining multiple biomarkers—such as Aβ, p-tau, and NfL—into a composite panel provides a more comprehensive understanding of AD pathology, improving diagnostic accuracy.

Recent research has also highlighted the critical roles of immune and inflammatory processes in AD pathogenesis, particularly involving microglia and astrocytes. Network-based analyses, such as those using the STRING database, have uncovered essential protein interactions that provide a broader understanding of the disease. Additionally, microRNA interaction networks are emerging as promising cumulative biomarkers for tracking AD progression.

Non-invasive brain stimulation techniques, such as transcranial magnetic stimulation and transcranial direct current stimulation, hold significant potential for diagnosing AD and offering therapeutic interventions. Studies by Freitas et al. [207] and Hall et al. [208] demonstrated distinct cortical activity and connectivity patterns that could serve as biomarkers for distinguishing AD from cognitively healthy individuals. Moreover, research by Koch et al. [209] highlighted transcranial magnetic stimulation as an emerging diagnostic tool for detecting early synaptic dysfunction, reinforcing the value of non-invasive brain stimulation in diagnostics and treatment.

However, challenges remain in validating BBBMs across diverse populations. Factors such as age, sex, genetics, and comorbidities can influence biomarker levels, contributing to variability in diagnostic performance. Additionally, the lack of insurance coverage and reimbursement in many regions limits clinical adoption. Nevertheless, BBBMs are becoming increasingly critical in resource-limited settings, where traditional diagnostics like PET and CSF analysis are less accessible. The development of scalable diagnostic tools holds great promise for early detection and disease monitoring, especially in low- to middle-income countries.

Looking ahead, research efforts should focus on standardizing BBBM assays, establishing universally accepted diagnostic cut-off values, and conducting large-scale studies across diverse populations to validate their diagnostic and prognostic utility. Integration with advanced neuroimaging techniques and artificial intelligence-driven analysis could further enhance the diagnostic power of BBBMs, offering more precise disease staging and monitoring. Additionally, the potential of BBBMs to identify asymptomatic individuals at risk of developing AD presents new opportunities for early intervention, which could ultimately slow disease progression and reduce the global AD burden.

In conclusion, while significant challenges remain, the ongoing development of BBBMs represents a crucial step toward more accessible, cost-effective, and accurate diagnostics for AD. As these biomarkers are further validated and integrated into clinical practice, they have the potential to transform the landscape of AD diagnosis and enable more personalized treatment strategies.

## Figures and Tables

**Table 1:** Overview of blood-based biomarkers in Alzheimer’s disease: assay techniques, pathomechanisms, and their role in diagnostics and prognostics.

Name of the Blood-Based Biomarkers		Underlying Pathophysiology	Categorization (NIA-AA 2024)	Relevance	Trend of the Biomarker in Plasma	Assessment Techniques
**Amyloid β** (**Aβ**)	Aβ42	Plasma biomarkers related to amyloid accumulation in AD	Core 1 biomarker (**A**)	Early detection of AD in asymptomatic individuals can facilitate the transition from normal cognition to mild cognitive impairment or AD	Decreased in AD and mild cognitive impairment compared to controls	Enzyme-linked immunosorbent assay (ELISA), Luminex xMAP Technology, single-molecule array (SIMOA), liquid chromatography-mass spectrometry, and immunoprecipitation mass spectrometry
	Aβ40	Plasma biomarkers related to amyloid accumulation in AD	Core 1 biomarker (**A**)	Early detection of AD in asymptomatic individuals can indicate progression from normal cognition to mild cognitive impairment or AD	Decreased in AD and mild cognitive impairment compared to controls	
	Aβ42/40	Plasma biomarkers related to amyloid accumulation in AD	Core 1 biomarker (**A**)	It can identify the early stages of AD and predict cognitive decline in accordance with cerebrospinal fluid and neuroimaging biomarkers	Decreased Aβ42/Aβ40 ratio in AD and mild cognitive impairment compared to controls	
**Tau**	p-tau217	Plasma biomarkers of phosphorylated and secreted tau in AD	Core 1 biomarker (**T_1_**)	Early detection of AD in asymptomatic individuals can accurately predict the progression from subjective cognitive decline and mild cognitive impairment to dementia when combined with other risk factors	Increased in AD and mild cognitive impairment compared to controls	ELISA, Luminex xMAP Technology, SIMOA, liquid chromatography-mass spectrometry, and immunoprecipitation mass spectrometry
	p-tau181	Plasma biomarkers of phosphorylated and secreted tau in AD	Core 1 biomarker (**T_1_**)	Early detection of AD in asymptomatic individuals distinguishes between Aβ-positron emission tomography (PET) positive and Aβ-PET negative individuals, as well as correlates with disease progression to dementia. This detection is associated with tau-burdened brain areas exhibiting AD-related atrophic changes	Increased in AD and mild cognitive impairment compared to controls	
	p-tau231	Plasma biomarkers of phosphorylated and secreted tau in AD	Core 1 biomarker (**T_1_**)	Early detection of AD) in asymptomatic individuals differentiates between patients with and without AD pathology during post-mortem assessment	Increased in AD and mild cognitive impairment compared to controls	
	MTBR-tau243	Plasma biomarkers related to tau accumulation in AD	Core 2 biomarker (**T_2_**)	Elevated in the later stages of AD, this biomarker, along with the Core 1 biomarker, is strongly associated with tau-PET imaging and disease progression, reflecting the staging of biological disease severity	Increased in AD and mild cognitive impairment compared to controls	
	Non-phosphorylated mid-region tau fragments	Plasma tau-related biomarkers associated with tau accumulation in AD	Core 2 biomarker (**T_2_**)	Elevated in the later stages of AD, the staging of biological disease severity is associated with the Core 1 biomarker	Increased in AD and mild cognitive impairment compared to controls	
**α-Synuclein**	α-Synuclein/tau	Biomarkers related to abnormal protein accumulation in non-core AD pathology, specifically synuclein pathology	Biomarkers of non-AD co-pathology (**S**)	Total α-synuclein levels in the blood may not differ significantly between patients with neurodegenerative diseases. However, the oligomeric or phosphorylated forms of α-synuclein are associated with accelerated cognitive dysfunction	Decreased in AD and mild cognitive impairment vs. controls	Seed amplification assays: Protein Misfolding Cyclic Amplification and Real-Time Quaking-Induced Conversion Detection techniques: ELISA, Western blotting, Quantitative Mass Spectrometry, Luminex xMAP Technology, Surface Plasmon Resonance–Dynamic Light Scattering, and Immuno-Polymerase Chain Reaction
	α-Synuclein/Aβ 42		Biomarkers of non-AD co-pathology		Increased in AD and mild cognitive impairment compared to controls	
**Dickkopf-1**		Biomarkers related to abnormal protein accumulation in non-core AD pathology	Research biomarker	Elevated levels correlate with disease severity, particularly cognitive decline, and synaptic loss, and help differentiate AD from other neurodegenerative conditions	Increased in AD	ELISA, Western blotting, Luminex xMAP Technology, Immuno-Polymerase Chain Reaction, and mass spectrometry
**Visinin-like protein-1** (**VILIP-1**)		Biomarkers related to abnormal protein accumulation in non-core AD pathology	Research biomarker	Increased levels are observed in AD; however, no significant differences in concentrations are found between AD-mild cognitive impairment patients and other neurodegenerative groups	Increased in AD	ELISA, Western blotting, Luminex xMAP Technology, Immuno-Polymerase Chain Reaction, and mass spectrometry
**Plasma neurofilament light chain** (**NfL**)		Injury, dysfunction, or degeneration of neuropil	Biomarkers of non-specific processes involved in AD pathophysiology (**N**)	Increased levels in Aβ-positive patients with AD and mild cognitive impairment are associated with the degree of cognitive impairment and are utilized as monitoring biomarkers to indicate the severity of neurodegeneration	Increased in AD and mild cognitive impairment compared to controls	ELISA, Luminex xMAP Technology, electrochemiluminescence immunoassay, mass spectrometry, and SIMOA
**Synaptosome-associated protein of 25 kDa** (**SNAP-25**)		Neuronal and synaptic injury related to presynaptic dysfunction.	Biomarkers of non-specific processes involved in AD pathophysiology (**N**)	Cerebrospinal concentrations can differentiate between various neurodegenerative diseases such as AD, Parkinson’s disease, and amyotrophic lateral sclerosis	Decreased in AD compared to controls	ELISA, Western blotting, Luminex xMAP Technology, Immuno-Polymerase Chain Reaction, and mass spectrometry
**Neuronal pentraxin 2** (**NPTX-2**)		Neuronal and synaptic injury related to presynaptic dysfunction.	Biomarkers of non-specific processes involved in AD pathophysiology (**N**)	It has the potential as a biomarker for the early detection of AD	Decreased in AD vs. controls	ELISA, Western blotting, Luminex xMAP Technology, Immuno-Polymerase Chain Reaction, and mass spectrometry
**Growth-associated protein 43** (**GAP-43**)		Neuronal and synaptic injury related to presynaptic dysfunction.	Biomarkers of non-specific processes involved in AD pathophysiology (**N**)	It has the potential as a biomarker for the early detection of AD	Increased in AD compared to controls	ELISA, Western blotting Luminex xMAP Technology, Immuno-Polymerase Chain Reaction, and mass spectrometry
**Neurogranin** (**NG**)		Neuronal and synaptic injury related to postsynaptic protein dysfunction	Biomarkers of non-specific processes involved in AD pathophysiology (**N**)	It has the potential as a biomarker for the early detection of AD	Decreased in AD vs. controls	ELISA, Luminex xMAP Technology, electrochemiluminescence immunoassay, mass spectrometry, and SIMOA
**Fms-like tyrosine kinase-1** (**Flt-1**)		Vascular damage related to AD	Research biomarker (**V**)	It assesses total vascular involvement and aids in the early detection of vascular changes associated with AD	Increased in AD compared to controls	ELISA, Western blotting, Luminex xMAP Technology, Immuno-Polymerase Chain Reaction, and mass spectrometry
**Endothelin 1** (**ET-1**)		Vascular damage related to AD	Research biomarker (**V**)	It reflects vascular impairment in AD	Increased in AD compared to controls	ELISA, Luminex xMAP Technology, Immuno-Polymerase Chain Reaction, and mass spectrometry
**Atrial natriuretic peptide** (**ANP**)		Vascular damage related to AD	Research biomarker (**V**)	It leads to reduced cerebral blood flow and impairment of neurovascular health	Increased in AD compared to controls	ELISA, Luminex xMAP Technology, Immuno-Polymerase Chain Reaction, and mass spectrometry
**Monokine induced by gamma interferon** (**MIG/CXCL9**)		Vascular damage related to AD	Research biomarker (**V**)	It indicates the presence of ongoing chronic neuroinflammatory processes	Increased in AD compared to controls	ELISA, Luminex xMAP Technology, Immuno-Polymerase Chain Reaction, and mass spectrometry
**Heart-type fatty acid-binding protein** (**H-FABP**)		Vascular damage related to AD	Research biomarker (**V**)	It shows potential as a probable biomarker for the early detection of AD, as elevated levels have been found in the preclinical phase of AD dementia	Increased in AD compared to controls	ELISA, Western blotting, Luminex xMAP Technology, Immuno-Polymerase Chain Reaction, and mass spectrometry
**Vascular Adhesion Molecule-1** (**VCAM-1**)	Soluble vascular cell adhesion molecule-1 (sVCAM-1)	Vascular damage related to AD	Research biomarker (**V**)	Elevated sVCAM levels indicate the burden of atherosclerosis in AD, showing a significant correlation between age and the severity of cognitive decline	Increased in AD compared to controls	ELISA, Western blotting, Luminex xMAP Technology, Immuno-Polymerase Chain Reaction, and mass spectrometry
	Soluble intercellular adhesion molecule-1 (sICAM-1)	Vascular damage related to AD	Research biomarker (**V**)	Elevated levels of sICAM-1 indicate the burden of atherosclerosis in AD	Increased in AD compared to controls	ELISA, Western blotting, Luminex xMAP Technology, Immuno- Immuno-Polymerase Chain Reaction, and mass spectrometry
**Metabolic products secondary to lipid peroxidation**	Malondialdehyde (MDA)	Oxidative stress	Research biomarker	Increased levels are observed in familial AD associated with mutations in the amyloid precursor protein and presenilin-1 genes	Increased in AD compared to controls	High-performance liquid chromatography, liquid chromatography-mass spectrometry, ELISA, and gas chromatography-mass spectrometry
	4-hydroxynonenal (HNE)	Oxidative stress	Research biomarker	Increased levels are observed in familial AD associated with mutations in the amyloid precursor protein and presenilin-1 genes	Increased in AD compared to controls	
	Increased F2-isoprostanes	Oxidative stress	Research biomarker	As a potential marker of oxidative stress during the mild cognitive impairment phase of AD, its levels correlate with the disease continuum, ranging from subjective cognitive decline to mild cognitive impairment and eventually to AD	Increased in AD compared to controls	
**Free radicals**	Reactive oxygen species	Oxidative damage	Research biomarker	Reactive oxygen species modify neuronal macromolecules and induce hyperphosphorylation of tau protein during the prodromal phases of AD	Increased in AD	Dichloro-fluorescein Diacetate Assay, Electron Spin Resonance Spectroscopy, Nitroblue Tetrazolium Assay, and Flow Cytometry with reactive oxygen species-sensitive dyes
	Reactive nitrogen species	Oxidative damage	Research biomarker	Nitrosylation of critical proteins in neurons impairs their function, promoting neurodegenerative processes	Increased in AD	Nitrotyrosine ELISA, Electron Spin Resonance Spectroscopy, and Western blot for 3-Nitrotyrosine-modified proteins
**Nucleoside 8-hydroxyguanosine** (**8-OHG**)		Oxidative damage	Research biomarker	It is significant for assessing the gradient of DNA oxidative damage in patients with AD, enabling the early detection of oxidative damage to plasma DNA	Increased in lymphocytes of AD patients compared to control	ELISA, high-performance liquid chromatography with electrochemical detection, liquid chromatography-mass spectrometry, Western Blot using specific anti-5.8-OHG antibodies, immunoprecipitation, and gas chromatography-mass spectrometry
**Mitochondrial respiratory complex I-V genes** (**OxPHOS genes**)		Bioenergetic abnormality	Research biomarker	An imbalance between nuclear and mitochondrial genome-encoded OXPHOS transcripts may trigger a negative feedback loop, reducing mitochondrial translation and compromising OXPHOS efficiency. This imbalance is likely to result in the increased generation of harmful reactive oxygen species	Reduced expression in early AD patients	Quantitative polymerase chain reaction, Western blot, Immunohistochemistry, and Blue Native Gel Electrophoresis
**S-nitrosylated dynamin-related protein 1** (**SNO-Drp1**)		Bioenergetic abnormality	Research biomarker	SNO-Drp1 can lead to increased mitochondrial fission, synapse loss, and neuronal damage in mouse models, primary neuronal cultures, and post-mortem tissue	Increased levels of SNO-Drp1 are observed in peripheral blood lymphocytes of patients with AD. However, there are contradictory findings indicating that SNO-Drp1 levels do not differ significantly between AD patients and controls	Biotin Switch Assay, mass spectrometry, Nitroso-Proteome Profiling, immunoprecipitation, and Western blot
**Mitochondrial DNA**		Bioenergetic abnormality	Research biomarker	Mitochondrial DNA copy number serves as an indirect indicator of mitochondrial function, providing valuable information about bioenergetics as a contributing factor in the progression of AD	Decreased in patients with AD	Quantitative polymerase chain reaction, digital droplet polymerase chain reaction, and Southern blotting
**8-oxo-7,8-dihydroguanine somatic single nucleotide variants** (**8oxoG sSNVs**)		Bioenergetic abnormality	Research biomarker	Due to its inflammatory endophenotype, the circulating cell-free mtDNA 8oxoG variant can be utilized as an enhanced biomarker	Increased in AD patients	8-oxoG DNA Glycosylase Assay, Comet Assay with Formamidopyrimidine-DNA Glycosylase, ELISA, and high-performance liquid chromatography with electrochemical detection
**Circulating cell-free mtDNA**		Bioenergetic abnormality	Research biomarker	Cellular mitochondrial DNA copy number can serve as a potential biomarker of mitochondrial biogenesis and cellular energetics, reflecting mitochondrial health in AD	Increased in AD patients	Quantitative polymerase chain reaction, digital droplet quantitative polymerase chain reaction, and Southern blotting
**Intermediate filament glial fibrillary acidic protein** (**GFAP**)		Neuroinflammation and immune dysregulation	Research biomarker (**I**)	Marker of astrogliosis observed in chronic inflammatory processes, such as in progressing AD	Increased in AD patients	ELISA, electrochemiluminescence immunoassay, and Mesoscale Discovery Immunoassay V-PLEX
**CX3CL1** (**Fractalkine**)		Neuroinflammation and immune dysregulation	Research biomarker (**I**)	Significantly elevated in the plasma of patients with mild cognitive impairment and AD compared to other neuroinflammatory disease processes	Increased in AD and MCI	ELISA, Western blot, Immunohistochemistry, Flow Cytometry, and Luminex
**C-C motif chemokine ligand 23** (**CCL23**)		Neuroinflammation and immune dysregulation	Research biomarker (**I**)	Their plasma concentration has also been found to have a predictive value for the progression from MCI to AD	Increased in AD	ELISA, Western blot, Immunohistochemistry, Flow Cytometry, and Luminex
**C-C chemokine ligands or regulated upon activation, normal T cell expressed and secreted** (**RANTES/CCL5**)		Neuroinflammation and immune dysregulation	Research biomarker (**I**)	Elevated in AD and correlated with the neuroinflammatory burden	Increased in AD	ELISA, Western blot, Immunohistochemistry, Flow Cytometry, and Luminex
**YKL-40**		Neuroinflammation and immune dysregulation	Research biomarker (**I**)	Increasingly expressed in astrocytes during neuroinflammatory changes, plasma YKL-40 levels have been shown to positively correlate with the results of the sensitive Free and Cued Selective Reminding Test	Increased in AD	ELISA, Western blot, Immunohistochemistry, Flow Cytometry, and Luminex
**Progranulin**		Neuroinflammation and immune dysregulation	Research biomarker (**I**)	Increased expression of the progranulin gene is found in the blood of patients with mild cognitive impairment and AD	Increased in AD	ELISA, Western blot, Immunohistochemistry, Flow Cytometry, and Luminex
**Triggering receptor expressed on myeloid cells 2** (**TREM2**)		Neuroinflammation and immune dysregulation	Research biomarker (**I**)	Messenger RNA levels in peripheral mononuclear cells have been found to distinguish between amnestic mild cognitive impairment, AD, and healthy control individuals and are dependent on the apolipoprotein E genotype	Increased in AD	ELISA, Western Blot, Immunohistochemistry, Flow Cytometry, and Luminex
**Neuronal-derived exosomes**	P-S396-tau	Tauopathy	Research biomarker	It can predict the development of AD up to 10 years before the clinical onset of sporadic AD	Increased in AD	Proteomic analysis of extracellular vesicles, such as through ELISA
	p-tau181	Tauopathy	Research biomarker	It has the potential to predict the development of AD up to 10 years before the clinical onset of sporadic AD	Increased in AD and mild cognitive patients compared to controls	ELISA and ultra-sensitive inhouse SIMOA
	Synaptotagmin	Synaptopathy	Research biomarker	Its impairment leads to decreased neurotransmission, neuroplasticity, and long-term potentiation, thus hampering memory formation	Reduced in AD	ELISA, liquid chromatography-mass spectrometry, and SIMOA
	Synaptophysin	Synaptic loss and dysfunction	Research biomarker	Loss of proper functioning synapse leads to impaired signal transmission and, thus, cognitive impairment	Reduced in AD	ELISA, liquid chromatography-mass spectrometry, and SIMOA
	Phosphorylation of insulin receptor substrate-1 (IRS-1) at serine 312 (P-S312-IRS-1)	Neuroinflammation and insulin resistance	Research biomarker	Its increment promotes insulin resistance, leading to progressive neurodegeneration	Increased in AD vs. controls	ELISA, liquid chromatography-mass spectrometry, and SIMOA
	Phosphorylation at multiple tyrosine residues of insulin receptor substrate-1 (P-panY-IRS-1)	Insulin resistance and synaptic dysfunction	Research biomarker	Its reduction promotes insulin resistance, leading to progressive neurodegeneration	Downregulated in AD	ELISA, liquid chromatography-mass spectrometry, and SIMOA
	N-(1-carboxymethyl)-L-lysine	Reactive oxygen species-mediated damage	Research biomarker	It can differentiate between the early and moderate stages of AD	Downregulated in AD	ELISA, liquid chromatography-mass spectrometry, and SIMOA
**Malondialdehyde**		Tauopathy	Research biomarker	When neurons absorb microglia-derived exosomes containing tau, it triggers additional abnormal tau aggregation	Increases in AD	ELISA and liquid chromatography-mass spectrometry
**Astrocyte-derived exosomes**		Neuroinflammation	Research biomarker	Plasma levels of various complement components, such as C1q, C3b, and factor D, could serve as predictive biomarkers for the progression of mild cognitive impairment to AD	Increases in AD	ELISA and liquid chromatography-mass spectrometry

**Table 2:** List of microRNAs identified in whole blood, plasma, serum, and peripheral blood mononuclear cells based on extensive literature search.

Source of the microRNA	Names of microRNA	Reference
Whole blood	hsa-miR-107	[161,162,163,165]
Plasma	hsa-miR-92a-3p, hsa-miR-486-5p, hsa-miR-29a-3p, hsa-miR-107, hsa-miR-128-3p, hsa-miR-132-3p, hsa-miR-34c-5p, hsa-let-7d-5p, hsa-miR-191-5p, hsa-miR-200a-3p, hsa-miR-483-5p, hsa-miR-486-5p, hsa-miR-502-3p, hsa-miR-548k, hsa-miR-339-5p, hsa-miR-221-5p, hsa-miR-144-5p, hsa-miR-382-5p, hsa-miR-146b-5p, hsa-miR-224-5p, hsa-miR-625-5p, hsa-miR-769-5p, hsa-miR-454-5p, hsa-miR-548d-5p, hsa-miR-877-5p, hsa-miR-146a, hsa-miR-125b,	
Serum	hsa-miR-106-b-3p, hsa-miR-22-3p, hsa-miR-126-5p, hsa-miR148b-5p, hsa-miR-181c-3p, hsa-miR-93-5p, hsa-miR-29c-3p, hsa-miR-132-3p, hsa-miR-222-3p, hsa-let-7d-5p, hsa-miR-191-5p, hsa-miR-146a, hsa-miR-125b, hsa-miR-135a	
Peripheral blood mononuclear cells	hsa-miR-128-3p, hsa-miR-34c-5p	

**Table 3:** Differential expression of BBBMs between hereditary and sporadic Alzheimer’s disease.

Biomarker	Hereditary AD (Early Onset)	Sporadic AD (Late Onset)
**Aβ42/Aβ40 Ratio**	Decreased earlier, often in the preclinical phase	Decreased later, closer to symptom onset
**P-tau181**	Increases 6–10 years before symptoms; correlates with Aβ pathology	Rises later but remains a strong marker of tau pathology
**T-tau**	Poor discriminatory power; less significant changes	Variable changes; lacks a strong association with AD progression
**NfL**	Increases closer to symptom onset; higher variability	Rises later; more consistent correlation with neurodegeneration
**GFAP**	Rises ten years before symptoms, indicating early astrocytic activation	Early rise, but slightly later than in hereditary forms

Aβ: amyloid-beta; P-tau181: phosphorylated tau at threonine 181; T-tau: total tau; NfL: neurofilament light chain; GFAP: glial fibrillary acidic protein; AD: Alzheimer’s disease.

**Table 4:** Differential expression of AD-related BBBMs in comparison to frontotemporal dementia, Lewy body dementia, and vascular dementia.

Dementia Type	GFAP	p-Tau181	NfL	T-Tau	Correlating Features
**AD**	Elevated early	Elevated	Elevated	No significant alteration	Correlates with Aβ, tau pathology
**FTD**	Elevated	Mildly elevated	Elevated	Elevated	p-Tau181 demonstrates higher diagnostic accuracy in distinguishing FTD from AD
**LBD**	Elevated	Mildly elevated	Elevated	Elevated	All biomarkers overlap with AD pathology
**VaD**	No significant alteration	No significant alteration	Elevated	No significant alteration	Correlates with the extent of neurovascular damage

GFAP: glial fibrillary acidic protein; p-Tau181: phosphorylated tau 181; NfL: neurofilament light chain; T-tau: total tau; AD: Alzheimer’s disease; FTD: frontotemporal dementia; LBD: Lewy body dementia; VaD: vascular dementia.

## Data Availability

Anonymized data will be made available upon reasonable request to qualified researchers. Requests should be directed to Dr. Julián Benito-León at jbenitol67@gmail.com.

## Abbreviations

AAO, age at symptom onset; ALCAM, activated leukocyte cell adhesion molecule; APP, amyloid precursor protein; Aβ, amyloid-beta; AD, Alzheimer’s disease; ADM, Adrenomedullin; ADNPC, Alzheimer’s Disease Neuropathological Changes; AGER, Advanced Glycosylation End-Product Specific Receptor; ALS, amyotrophic lateral sclerosis; AM, adhesion molecule; ANP, atrial natriuretic peptide; APOCIII, Apolipoprotein CIII; AT(N), Amyloid/Tau/Neurodegeneration; α-syn, α-synuclein; BACE1, Beta-Secretase 1; BACE2, Beta-Secretase 2; BBBMs, blood-based biomarkers; CCL, C-C motif chemokine ligand; CF-mt-DNA, Cell-Free Mitochondrial DNA; CircRNA, Circular RNA; COX20, Cytochrome C Oxidase Subunit 20; COX5A, Cytochrome C Oxidase Subunit 5A; COX6A2, Cytochrome C Oxidase Subunit 6A2; COX8A, Cytochrome C Oxidase Subunit 8A; CSF, cerebrospinal fluid; DKK1, Dickkopf-Related Protein 1; DLS, Dynamic Light Scattering; DNM1L, Dynamin 1-Like; ELISA, Enzyme-Linked Immunosorbent Assay; EOAD, early-onset Alzheimer’s disease; ET-1, Endothelin 1; EV, exosomal vesicles; FABP3, Fatty Acid-Binding Protein 3; FDG, Fluorodeoxyglucose; Flt-1, Fms-like tyrosine kinase-1; FTD, frontotemporal dementia; GAP-43, growth-associated protein 43; GC-MS, gas chromatography–mass spectrometry; GFAP, glial fibrillary acidic protein; H-FABP, heart-type fatty acid-binding protein; HPLC, high-performance liquid chromatography; IFN-γ, interferon-gamma; IHC, Immunohistochemistry; IL, interleukin; Immuno-PCR, Immuno-Polymerase Chain Reaction; IP-MS, immunoprecipitation–mass spectrometry; LC-MS, liquid chromatography–mass spectrometry; lncRNA, Long Non-Coding RNA; MAPT, microtubule-associated protein tau; MCI, mild cognitive impairment; MIG/CXCL9, monokine induced by gamma interferon/C-X-C motif chemokine ligand 9; MMSE, Mini-Mental State Examination; MRI, magnetic resonance imaging; MT-ND1, Mitochondrially Encoded NADH Oxidoreductase Core Subunit 1; MT-ND2, Mitochondrially Encoded NADH Oxidoreductase Core Subunit 2; MT-ND3, Mitochondrially Encoded NADH Oxidoreductase Core Subunit 3; MT-ND4, Mitochondrially Encoded NADH Oxidoreductase Core Subunit 4; MT-ND5, Mitochondrially Encoded NADH Oxidoreductase Core Subunit 5; NDUFA4, NADH Oxidoreductase Subunit A4; NIA-AA, National Institute on Aging–Alzheimer’s Association; NDUFS3, NADH Oxidoreductase Iron-Sulfur Protein 3; NfL, neurofilament light chain; NG, neurogranin; NOX1, NADPH oxidase 1; NOX3, NADPH oxidase 3; NOX4, NADPH oxidase 4; NPTX-2, neuronal pentraxin 2; PET, positron emission tomography; PRKN, Parkin RBR E3 Ubiquitin Protein Ligase; RBCs, red blood cells; SIMOA, single-molecule array; SNAP25, synaptosome-associated protein 25; SNCA, Synuclein Alpha; SPR, Surface Plasmon Resonance; sST2, Soluble Form of the Interleukin 33 Receptor; sVCAM-1, soluble vascular cell adhesion molecule-1; STRING, Search Tool for the Retrieval of Interacting Genes/Proteins; TDP-43, TAR DNA-Binding Protein 43; TREM1, triggering receptor expressed on myeloid cells 1; TREM2, triggering receptor expressed on myeloid cells 2; VCAM1, vascular cell adhesion molecule; VILIP-1, visinin-like protein 1; V-PLEX, Validated Plasma Exchange; YKL-40, Chitinase 3-Like 1.
